# Biofilm-isolated *Listeria monocytogenes* exhibits reduced systemic dissemination at the early (12–24 h) stage of infection in a mouse model

**DOI:** 10.1038/s41522-021-00189-5

**Published:** 2021-02-08

**Authors:** Xingjian Bai, Dongqi Liu, Luping Xu, Shivendra Tenguria, Rishi Drolia, Nicholas L. F. Gallina, Abigail D. Cox, Ok-Kyung Koo, Arun K. Bhunia

**Affiliations:** 1 Molecular Food Microbiology Laboratory, Department of Food Science, Purdue University, West Lafayette, IN USA; 2 Purdue Institute of Inflammation, Immunology and Infectious Disease, Purdue University, West Lafayette, IN USA; 3 Department of Comparative Pathobiology, Purdue University, West Lafayette, IN USA; 4 Department of Food and Nutrition, Gyeongsang National University, Jinju, Republic of Korea; 5 Institute of Agriculture and Life Science, Gyeongsang National University, Jinju, Republic of Korea

**Keywords:** Pathogens, Biofilms, Environmental microbiology

## Abstract

Environmental cues promote microbial biofilm formation and physiological and genetic heterogeneity. In food production facilities, biofilms produced by pathogens are a major source for food contamination; however, the pathogenesis of biofilm-isolated sessile cells is not well understood. We investigated the pathogenesis of sessile *Listeria monocytogenes* (*Lm*) using cell culture and mouse models. *Lm* sessile cells express reduced levels of the *lap*, *inlA, hly*, *prfA*, and *sigB* and show reduced adhesion, invasion, translocation, and cytotoxicity in the cell culture model than the planktonic cells. Oral challenge of C57BL/6 mice with food, clinical, or murinized-InlA (InlA^m^) strains reveals that at 12 and 24 h post-infection (hpi), *Lm* burdens are lower in tissues of mice infected with sessile cells than those infected with planktonic cells. However, these differences are negligible at 48 hpi. Besides, the expressions of *inlA* and *lap* mRNA in sessile *Lm* from intestinal content are about 6.0- and 280-fold higher than the sessle inoculum, respectively, suggesting sessile *Lm* can still upregulate virulence genes shortly after ingestion (12 h). Similarly, exposure to simulated gastric fluid (SGF, pH 3) and intestinal fluid (SIF, pH 7) for 13 h shows equal reduction in sessile and planktonic cell counts, but induces LAP and InlA expression and pathogenic phenotypes. Our data show that the virulence of biofilm-isolated *Lm* is temporarily attenuated and can be upregulated in mice during the early stage (12–24 hpi) but fully restored at a later stage (48 hpi) of infection. Our study further demonstrates that in vitro cell culture assay is unreliable; therefore, an animal model is essential for studying the pathogenesis of biofilm-isolated bacteria.

## Introduction

*Listeria monocytogenes* (*Lm*) is a Gram-positive facultative intracellular pathogen causing listeriosis, notorious for its high fatality (20–30%) among immunocompromised individuals, such as the elderly (65 and older), pregnant women, infants, and the AIDS patients^1^. A recent study also showed individuals with damaged intestinal microbiota due to antibiotics or chemotherapy are at higher risk since the commensal microbes are considered the first line of defense against *Lm* infection^2^. During foodborne infection, *Lm* crosses the gut barrier utilizing *Listeria* adhesion protein (LAP), Internalin A (InlA), and M cells^3,4^. *Lm* LAP interacts with its cognate epithelial receptor, heat-shock protein 60 (Hsp60)^5–7^, and activates NF-κB and myosin light chain kinase (MLCK) to disrupt epithelial tight junction barrier for bacterial passage into the lamina propria during the early stage (24–48 h) of infection^3,8^. The pathogen also uses InlA for epithelial cell invasion and gut barrier crossing by transcytosis^9^, which plays a significant role possibly at the later stage of infection (72–96 h) on a mouse model of infection^4,10^. Another invasion protein, InlB, also promotes *Lm* invasion of hepatic and intestinal epithelial cells^11^. After cell invasion, the vacuole-trapped bacterium escapes into the cytoplasm with the aid of listeriolysin O (LLO, encoded in *hly*) and phospholipases (PlcA and PlcB), suppresses cellular proinflammatory response using internalin C (InlC), and moves from cell-to-cell by polymerizing host actin protein (ActA)^12–14^. *Lm* also survives in the vacuole for an extended period prompting latent infection^15^. The protein regulatory factor (PrfA) regulates expression of virulence genes (*hly, plc, actA*) located on the *Listeria* pathogenicity island necessary for intracellular survival and spread^16^ while stress response regulator, SigB, regulates virulence genes and other accessory genes required for bacterial survival in the harsh environment of food and the host gut^17,18^.

*Lm* existence is ubiquitous in water and earth and can form biofilm on the food-contact surface and food production environment; thus, biofilm serves as a potential source for contamination and threatens public food safety^19–23^. Evolutionarily, *Lm* is well equipped to make the transition from soil/plant/environment-living saprophytic lifestyle to an infective intracellular lifestyle in the human host^24^.

Biofilm formation is an essential survival strategy for bacteria by which they manage to colonize on a solid surface, absorb nutrients, proliferate, and communicate with other species through quorum sensing^25–27^. Furthermore, biofilm formation is also associated with the majority of human infections^28,29^. Biofilm is generally made up of bacterial cells and extracellular polymeric substances composed of polysaccharide, protein, eDNA, and other inorganic molecules^30,31^. In a biofilm, bacteria are physically protected from harmful environmental factors, for instance, antibiotics, acid or alkali, UV radiation, and osmotic stress^32,33^. Not only surviving in the niche, but bacteria could also be released from biofilms after they are matured^30^. Therefore, as long as *Lm* forms a biofilm on a food-contact surface, it could become a consistent contamination source. It has been reported that *Lm* strains with the same pulsotypes have been isolated from a food processing plant multiple times throughout a year^25^. Previously, multiple studies have observed significant differences in gene expression between sessile and planktonic *Lm* cells^34–36^ especially, the reduced expression of InlA, InlC, and LLO in biofilm cells^35,37^. However, none of them examined the pathogenicity of biofilm cells using cell culture or animal models. Therefore, the question arose - how infective are these *Lm* sessile cells from the biofilm, if a food is consumed immediately after being contaminated with these cells? In addition, can a conventional mammalian cell culture model^38^ that is used routinely in the laboratory predict the nature of infectivity of biofilm isolates accurately? Is there any direct correlation of in vitro infectivity data for biofilm-isolated cells with in vivo animal experimental data?

To date, various studies have reported the persistence and resistance of *Lm* cells in the biofilm to environmental stress and significant change in global gene expression^39,40^; however, the exact virulence attributes of *Lm* isolated from biofilm has not been fully elucidated. The objective of this study was to assess and compare the pathogenicity of biofilm-isolated and planktonic *Lm* cells using in vitro intestinal epithelial cell culture model and an in vivo mouse (C57BL/6) model at different stages of infection (12, 24, and 48 h). Besides, we also analyzed the expression of key virulence proteins (LAP and InlA) that are involved during the early stage of infection^4^ and the regulatory genes, *prfA* and *sigB*. The knowledge gained would help understand the pathogenesis and develop an intervention strategy for controlling biofilm-forming bacterial pathogens from causing infection.

## Results

### Food-isolated *L. monocytogenes* strains have higher biofilm-forming capability than clinical isolates

**Fig. 1:**
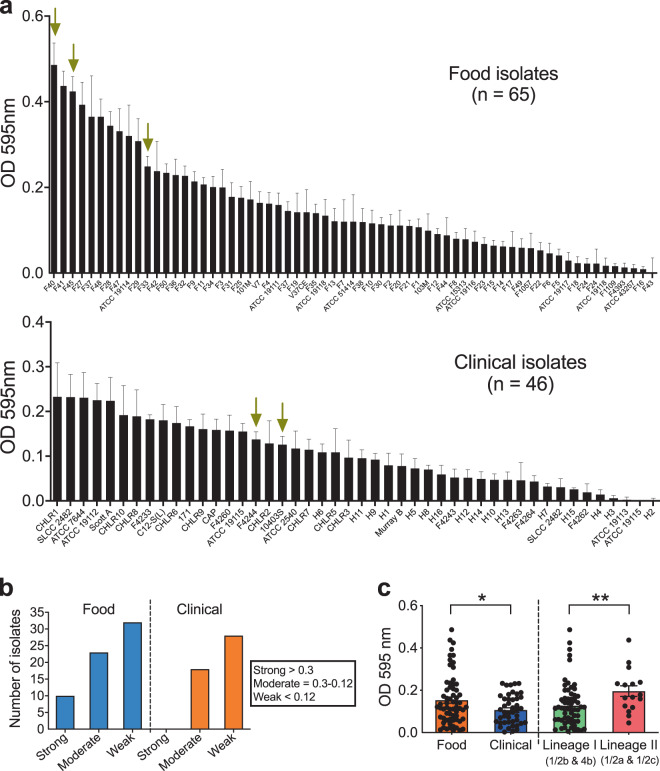
Quantification of *L. monocytogenes* biofilm formation and morphological analysis. **a** The biofilm-forming capabilities of over 100 food-(top panel) or clinical-isolated (bottom panel) *L. monocytogenes* (*Lm*) strains were tested using crystal violet staining assay. Arrows indicate the strains selected for further characterization. **b** Assemblage (strong, moderate, and weak) of isolates based on their ability to form biofilms. **c** Comparison of biofilm formation by food and clinical isolates and isolates of lineage I and II. Food isolates have significantly higher biofilm-forming capability than the clinical isolates, and isolates of lineage II also have a significantly higher capacity than isolates of lineage I. Mann–Whitney test was used for statistical analysis. **P < 0.005; *P < 0.05.

The biofilm-forming capability of over 100 *Lm* isolates of food and clinical origin on polystyrene surface was assessed after 48 h using crystal violet (CV) staining^41^ to choose for representative food and clinical strains to investigate the pathogenic potential in in vitro cell culture and in vivo mouse models. Biofilm-forming capacity varied widely among the strains at 48 h. We found food isolates (65 strains) had significantly (*P* < 0.05) higher biofilm-forming capability than the clinical isolates (46 strains) (Fig. 1a–c). Furthermore, isolates of serotype 1/2a and 1/2c (Lineage II) had greater biofilm-forming capabilities than serotypes 1/2b or 4b (Lineage I) (Fig. 1a, c). Isolates were arbitrarily grouped into high, moderate, and weak biofilm-producing groups (Fig. 1b) and representative strains with high (F40 and F45) or moderate (F33, F4244, and 10403S) biofilm-forming capabilities were chosen for further characterization. These isolates (two clinical: F4244 (4b) and 10403 S 1/2a) and three food: F40 (4b), F45 (1/2b), and F33 (4b)) represent serotypes 4b, 1/2b, and 1/2a (Supplementary Table S1) which are responsible for a majority of human listeriosis cases^1,42^. Light microscopic images revealed the formation of typical honeycomb-like structures of biofilms consistent with the previous observation^43^ with varying sizes of the biofilm clusters (Supplementary Fig. 1a). Furthermore, there is no apparent difference in individual cell lengths between sessile and planktonic cells (Supplementary Fig. 1b). Collectively, these data reveal that food-isolated *Lm* strains have higher biofilm-forming capabilities than clinical isolates.

### Biofilm-isolated *L. monocytogenes* has attenuated adhesion, invasion, and translocation capability to intestinal epithelial cell lines in vitro

**Fig. 2:**
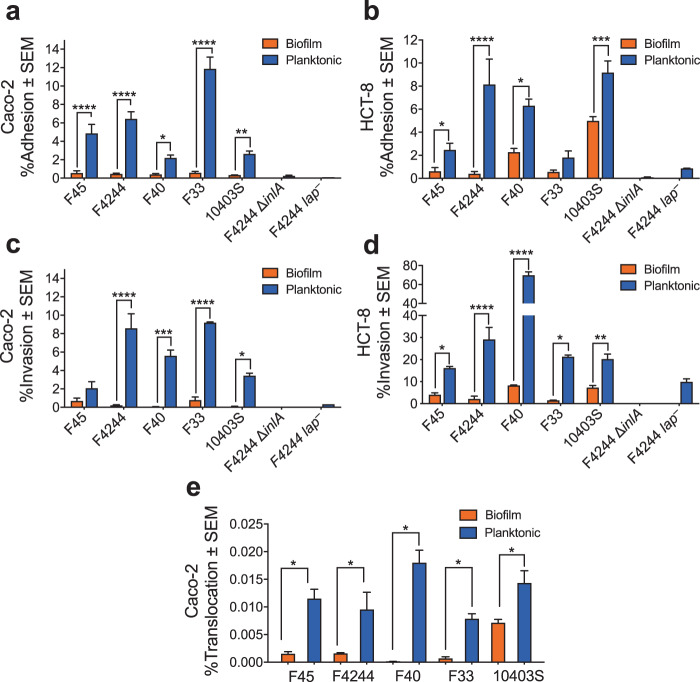
Adhesion, invasion, and translocation characteristics of biofilm-forming sessile and planktonic cells on the cultured cell line. Comparison of adhesion (**a, b**) and invasion (**c, d**) in Caco-2 and HCT-8 cells and transepithelial translocation across Caco-2 cells (**e**) between *L. monocytogenes* biofilm-isolated sessile and planktonic cells on Caco-2 and HCT-8 cells. The percentage was calculated by dividing the amounts of adhered, invaded, or translocated bacteria by the amounts of bacteria in the inoculum. Data are the average of at least three independent experiments performed in triplicate. All error bars represent SEM. Pairwise Student’s *t*-test was used for statistical analysis. ****P < 0.0001; ***P < 0.0005; **P < 0.005; *P < 0.05.

To compare the bacterial adhesion, invasion, and transmigration characteristics of 48-h-old biofilm-isolated sessile and 24-h-old planktonic *Lm* cells, two human intestinal epithelial cell lines, Caco-2 (colonic cells) and HCT-8 (Ileocecal junctional cells) were used. Sessile cells of all five strains (F40, F45, F33, F4244, and 10403S) tested showed a significantly (*P* < 0.05) decreased adhesion to Caco-2 (Fig. 2a) and HCT-8 (Fig. 2b) cells than that of their planktonic counterparts. Likewise, sessile cells showed significantly lower invasion than the planktonic cells into Caco-2 (Fig. 2c) and HCT-8 (Fig. 2d) cells. Sessile cells also showed significantly (*P* < 0.05) lower transepithelial translocation than the planktonic cells in a Transwell setup (Fig. 2e). Altogether, reduction in adhesion, invasion, and transepithelial migration between sessile and planktonic cultures was over 50%. Additionally, planktonic cells of wild-type (WT) *Lm* F4244 strain showed significantly higher adhesion and invasion than that of the planktonic cells of an isogenic *lap*^*─*^ or *∆inlA* mutant strains used as controls (Fig. 2). Note, during this experiment, the growth of both sessile and planktonic *Lm* cells were negligible in mammalian cell culture medium (D10F; Dulbecco’s modified Eagle’s medium (DMEM) with 10% fetal bovine serum) after 3 h and there is no significant difference (*P* > 0.05) between two cultures (Supplementary Fig. 2), suggesting the differences in bacterial interaction with mammalian cells are not influenced by their growth during the assay period. Taken together, these in vitro results suggest that the biofilm-isolated *Lm* strains have impaired ability to adhere, invade, or translocate across the epithelial cells than that of their planktonic counterparts thus possibly have reduced virulence potential.

### Biofilm-isolated *L. monocytogenes* were less cytotoxic to Ped-2E9 and Caco-2 cells than the planktonic bacteria

**Fig. 3:**
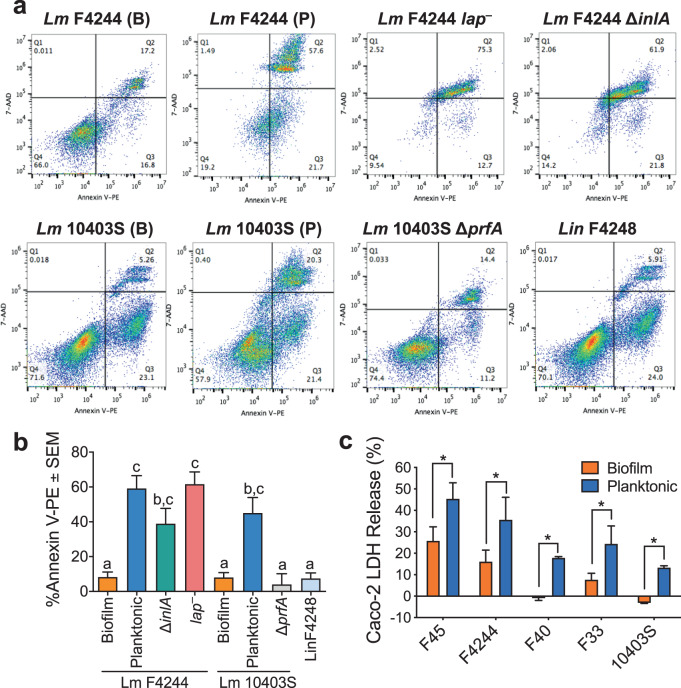
Cytotoxicity assessment of biofilm-forming sessile and planktonic *L. monocytogenes*. **a** Flow cytometric analysis of Ped-2E9 (B cell hybridoma) cells treated with biofilm-isolated (B) and planktonic (P) cells of *L. monocytogenes* (*Lm)* F4244 and 10403S and corresponding mutant strains at MOI 10. Annexin V-PE-positive and 7-AAD-negative events (Q3) were identified as cells in the early phase of apoptosis. Events with both Annexin V-PE and 7-AAD positive (Q2) or both negative (Q4) were identified as dead or live cells, respectively. *L. innocua* (*Lin*) F4248 was also tested as a nonpathogenic negative control. **b** Quantitative comparison of overall damage of Ped-2E9 caused by bacteria. Each bar represents the percentage of Annexin V-PE-positive events, which included early apoptosis (Q3) or dead (Q2) cells. Biofilm-isolated bacteria of both strains were significantly less cytotoxic than their planktonic counterparts. Bars marked with different letters are significantly different at *P* < 0.05. **c** Lactate dehydrogenase (LDH) released from Caco-2 cells (a colorectal adenocarcinoma) treated with both sessile or planktonic cells. Bacteria were incubated with cells at MOI 10 at 37 °C for 2 h. Data are the average of at least three independent experiments performed in triplicate. All error bars represent SEM. A pairwise Student’s *t*-test was used for statistical analysis. *P < 0.05.

To characterize pathogenic attributes of biofilm-isolated cells, Ped-2E9 (a hybrid murine B lymphocyte line)-based in vitro cytotoxicity assay was conducted^44,45^. Ped-2E9 has been established to be a sensitive model to respond to the apoptosis triggered by *Lm*^46–48^. We used Annexin V and 7-AAD labeling method to distinguish Ped-2E9 cells in the early or late apoptosis and analyzed them using flow cytometry and fluorescence microscopy. Biofilm-isolated and planktonic cells of *Lm* strains F4244 and 10403S and their corresponding isogenic mutant strains were analyzed (Fig. 3a). The damage caused by bacteria to Ped-2E9 cells was quantitatively compared by the sum percentage of Annexin V-positive events, which include cells in the early and the late stage (dead) of apoptosis (Supplementary Fig. 3). Firstly, all planktonic WT strains (F4244, and 10403S) caused significantly more cell damage than the corresponding biofilm-isolated cells (Fig. 3a, b). Secondly, the microscopic analysis confirmed that the planktonic strains (F4244, 10403S, and F45) are responsible for more apoptotic or dead Ped-2E9 cells than the sessile cells. Thirdly, as expected, planktonic 10403S cells caused significantly more damage than the isogenic *∆prfA* mutant strain whose virulence cannot be upregulated by the major regulator, PrfA^16^ (Fig. 3a, b). We used the *∆prfA* mutant as a negative control since *hly*, *plcA*, and *plcB* are regulated by PrfA and whose gene products are responsible for Ped-2E9 cell membrane damage and cytotoxicity^48,49^ Fourthly, F4244 *∆inlA* and *lap*^*─*^ mutant strains did not show a significant difference in cell death than that of the WT planktonic cultures, suggesting the cytotoxicity reduction in biofilm-isolated bacteria was not affected by InlA or LAP (Fig. 3a, b). Finally, *L. innocua* F4248, a nonpathogenic strain, did not induce any cytotoxicity (Fig. 3a, b). In addition, using Caco-2 cells as a second model, we observed planktonic cultures of five WT *Lm* strains (F45, F4244, F40, F33, and 10403S) to induce a significantly more lactate dehydrogenase (LDH) release than the sessile cultures, suggesting that planktonic *Lm* cells are more cytotoxic than the sessile cells (Fig. 3c). In sum, Ped-2E9 and Caco-2-based in vitro cytotoxicity data were consistent with the observation that biofilm-isolated bacteria have attenuated virulence compared to the planktonic bacteria on cultured cell lines.

### Key virulence factors were downregulated in biofilm-isolated bacteria on both transcription and translation levels

**Fig. 4:**
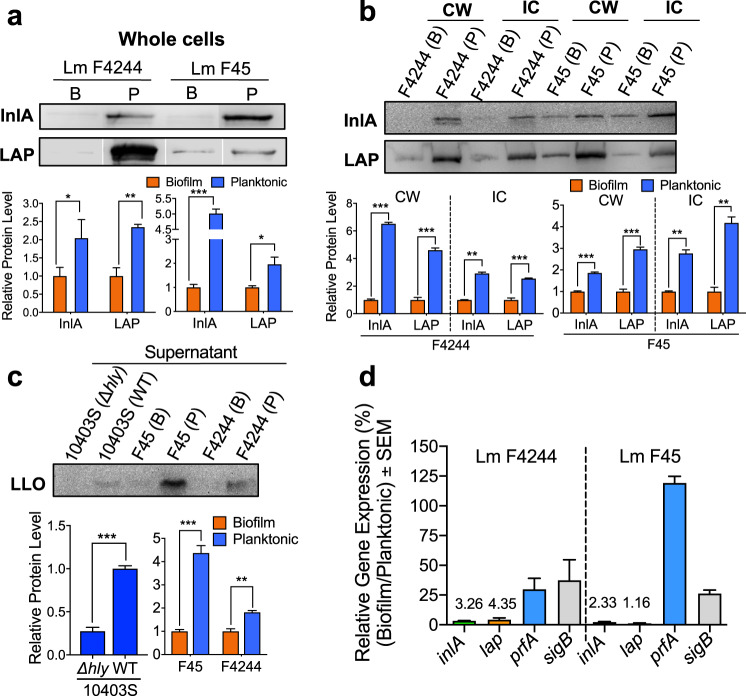
Comparison of virulence protein expression in biofilm-isolated (B) and planktonic (P) *L. monocytogenes* cells. **a**, **b** Immunoblot (top panel) and densitometry (bottom panels) of InlA, and LAP, in the whole cell (**a**), cell wall, and intracellular fractions (**b**) of biofilm-isolated and planktonic cultures of *Lm* F4244 and F45. Immunoblots are representative of three independent experiments. **c** Immunoblot of LLO in the secreted protein fraction of biofilm-isolated and planktonic *Lm* F4244 and F45. Immunoblots are representative of three independent experiments. **d** Relative mRNA expression of virulence genes (*inlA* and *lap*) and virulence regulators (*prfA* and *sigB*) in biofilm-isolated and planktonic cells of F4244 and F45 by RT-PCR. The Student’s *t*-test was used for statistical analysis. ***P < 0.0005; **P < 0.005; *P < 0.05.

Next, to unravel the underlying reduced in vitro adhesion, invasion, translocation, and cytotoxic phenotypes in sessile cells, we assessed the expression of mRNA and protein of key virulence factors using reverse transcriptional PCR and western blot in two representative strains: F4244 (clinical) and F45 (food). Western blot data showed that in biofilm-isolated cells, LAP, and InlA levels were all downregulated in the whole cells (Fig. 4a). Proteins in bacterial cells could be asymmetrically distributed in cytosol and cell wall, and the virulence molecules expressed on cell surface are responsible for interacting with epithelial cells. Therefore, we specifically compared the amount of those proteins in different cellular fractions. In cell wall and intracellular fractions, the amount of InlA and LAP were all significantly reduced in biofilm-isolated cells compared to those in the planktonic cells (Fig. 4b). At the same time, biofilm-isolated cultures also secreted significantly (*P* < 0.05) lower LLO in the supernatant compared to the planktonic cultures for strains F4244 and F45 (Fig. 4c). As a control for the anti-LLO antibody, the planktonic culture of 10403S showed a positive reaction with secreted LLO while an isogenic *∆hly* strain was negative (Fig. 4c). The protein samples were standardized with bicinchoninic acid assay method before loading onto the SDS-PAGE gel (Supplementary Fig. 4a).

To quantify the transcription of the major virulence genes, we first generated the standard curves for each gene’s copy number and Ct values and validated the specificity of qPCR primers. Standard curves for genes with copy numbers of approximately 10^1^–10^9^ copy/μL and Ct values were generated with each qPCR primer sets (Supplementary Table 2). All the standard curves had *R*^2^ values greater than 0.99 and a similar slope between −3.04 and −3.40 (Supplementary Fig. 4b, c). Besides, each primer pairs amplicon showed a sharp and single-peak melting curve (Supplementary Fig. 4d) suggesting that the qPCR primers are suitable for quantifying the target genes.

The gene-specific mRNA expression analysis in F4244 and F45 strain showed dramatic downregulation of both *lap* and *inlA* (~95–99%) in biofilm-isolated cells than that of the planktonic cells while both regulatory genes, *prfA*, and *sigB*, were downregulated by about 25% in the biofilm-isolated cells, except for the *prfA* in F45 (Fig. 4d). Compared to planktonic F45 cells, the amount of *prfA* mRNA is almost similar in biofilm-isolated F45 cells (Fig. 4d). These data suggest that the attenuated adhesion, invasion, transepithelial translocation, and cytotoxicity of biofilm-isolated *Lm* cells were possibly due to low expression of corresponding virulence genes at both mRNA and protein levels.

### In mouse bioassay, biofilm-isolated *Lm* showed reduced tissue burden at the early stage of infection (12–24 h), but similar to planktonic bacteria after 48 h post-infection

In vitro cell culture experiments suggest that biofilm-isolated *Lm* irrespective of food or clinical sources have a significantly lower capacity to adhere, invade, and translocate across the intestinal epithelial cells and lower cytotoxicity on B-lymphocytes and Caco-2 cells than the planktonic bacteria. However, the actual virulence of *Lm* in these two physiological states (sessile vs planktonic) cannot be accurately compared without observing their pathogenicity in vivo. Therefore, we orally infected 8–10 weeks old male and female C57BL/6 mice with both biofilm-isolated or planktonic *Lm* strains representing clinical (F4244) and food (F45) isolates with 1 × 10^9^ CFU/mouse and analyzed intestinal and extra-intestinal tissues for bacterial dissemination at 12, 24, and 48 h post-infection (hpi).

At 12 hpi, the bacterial burden in mice tissues was below the detection limit when a standard plating method was used. Therefore, we enriched the tissue samples in buffered *Listeria* enrichment broth (BLEB for 24 h) followed by isolation on the Modified Oxford (MOX) agar plate to determine the presence or absence of *Lm* in mice tissues. The select isolates were further verified by qPCR assay (Supplementary Table 3). To test the sensitivity of the detection method, we inoculated *Lm* (F4244 or F45) at 1.4 ± 0.2 or 1.2 ± 0.2 CFU/ml BLEB, respectively, and incubated at 37 °C for 24 h. Aliquots (10 µl) of each culture was streaked on MOX plates, and colonies with the black center were further verified by PCR, suggesting the two-step selective enrichment combined with PCR can detect approximately 1 CFU/ml of *Lm* in BLEB (Supplementary Fig. 5). None of the jejunal or ileal tissues of mice (*n* = 4–6) were positive for *Lm* after challenge with the sessile or planktonic cells (Table 1). However, only one of five (20%) cecum or colonic tissues were positive when mice were challenged with F4244 sessile cells compared to 50–100% positive when mice were challenged with the planktonic cells (Table 1). Likewise, no *Lm* cells were detected from the cecum and colon of mice when challenged with F45 sessile bacteria, whereas 80% (4/5) and 100% (5/5) were positive when challenged with planktonic bacteria, respectively (Table 1). Analysis of extra-intestinal organs/tissues; mesenteric lymph nodes (MLN), liver, and spleen of mice revealed that all the animals (*n* = 5) receiving F4244 sessile cells were negative while 16–50% mice (*n* = 6) were positive when infected with the planktonic cells (Table 1). Similarly, all mice receiving F45 sessile cells were negative in extra-intestinal organs, while 20–60% mice were positive when receiving planktonic cells (Table 1). None of the blood or kidney samples were positive when infected with both sessile or planktonic cells for both *Lm* strains at this early stage of infection. Nevertheless, these data indicate that bacterial intestinal invasion and subsequent systemic dissemination was lower for sessile cells than the planktonic cells for both *Lm* strains in mice after 12 hpi.

**Table 1:** *Listeria monocytogenes* strains (F45, F4244, and F4244 InlA^m^) translocation in C57BL/6 mice organs/tissues 12 h after oral infection.

Source	Tissues	Number of mouse tissues positive for *L. monocytogenes*/# mouse tested (%)^a^					
		F4244 (WT)		F45 (WT)		F4244 (InlA^m^)^b^	
		Biofilm	Planktonic	Biofilm	Planktonic	Biofilm	Planktonic
Intestinal	Jejunum	0/5 (0)	0/4 (0)	0/5 (0)	0/5 (0)	3/9 (33)	5/9 (56)
	Ileum	0/5 (0)	0/4 (0)	0/5 (0)	0/5 (0)	1/9 (11)	2/9 (22)
	Cecum	1/5 (20)	4/4 (100)	0/5 (0)	4/5 (80)	1/9 (11)	6/9 (67)
	Colon	1/5 (20)	2/4 (50)	0/5 (0)	5/5 (100)	2/9 (22)	5/9 (57)
Extra-Intestinal	MLN	0/5 (0)	3/6 (50)	0/5 (0)	2/5 (40)	0/9 (0)	2/9 (22)
	Liver	0/5 (0)	3/6 (50)	0/5 (0)	2/5 (20)	2/9 (22)	5/9 (56)
	Spleen	0/5 (0)	1/6 (16)	0/5 (0)	3/5 (60)	0/9 (0)	5/9 (56)
	Kidney	0/5 (0)	0/6 (0)	0/5 (0)	0/5 (0)	0/9 (0)	0/9 (0)

^a^Mice (both male and female) were orally gavaged with 1 × 10^9^ CFU/mouse. Mouse tissue samples were enriched in buffered *Listeria* enrichment broth for 24 h, plated on modified Oxford agar plate for 48 h, and 1–2 colonies per sample were verified by qPCR (see Supplementary Table 2).

^b^These animals received streptomycin (5 mg/ml) in water for 32 h, followed by 16 h antibiotic-free water before oral gavage with *Lm*.

**Fig. 5:**
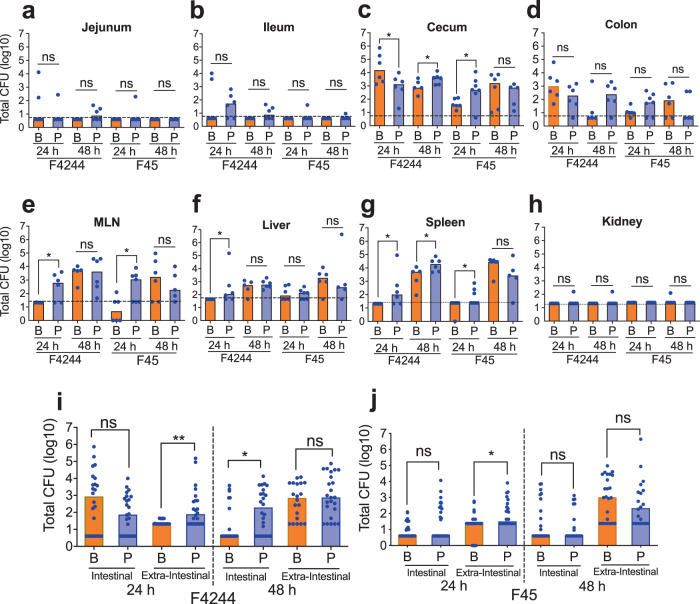
Mouse bioassay to compare the pathogenesis of biofilm-isolated sessile and planktonic *L. monocytogenes* cells. *Lm* burden in intestinal (**a**–**d**) and extra-intestinal tissues (**e**–**h**) after oral inoculation of mice (C57BL/6, male–female, 8–10 weeks old) with 1 × 10^9^ CFU/mouse of sessile (B) and planktonic (P) cells of F4244 or F45 at 24 and 48 hpi. **i**, **j** Comparison of the number of bacteria in all intestinal and extra-intestinal tissues at 24 and 48 hpi. Bars represent the median values of each group (B or P). Dashed lines indicate detection limits by a plating method. Mann–Whitney test was used for statistical analysis. **P < 0.005; *P < 0.05.

At 24 hpi, we were able to enumerate *Lm* in most mice organs and tissues by a standard plating method. In the intestinal tissues, there were no significant differences in bacterial counts between sessile and planktonic cells-challenged mice with an exception of the cecum, where sessile cells had significantly (*P* < 0.05) higher colonization than the planktonic cells (Fig. 5a–d). However, in the extra-intestinal organs (MLN, spleen, and liver) planktonic cells exhibited significantly (*P* < 0.05) higher bacterial burdens than the sessile cells (Fig. 5e–g). In fact, F4244 sessile cells were undetectable in these organs as determined by a plating method, suggesting that the sessile cells were either unable or translocated and/or disseminated in blood/lymphatic circulation at levels that are below our detection limits at 24 hpi. In the kidney, counts for both sessile and planktonic cells were below the detection limit with an exception of one mouse, which was showing the planktonic burden of about two logs (Fig. 5h). Altogether, planktonic F4244 cell-challenged mice had significantly (*P* < 0.005) higher total *Lm* burden than the sessile cell-challenged mice in the extra-intestinal organs while there was no significant difference in total bacterial burden in whole intestinal tissues combined at 24 hpi (Fig. 5i). Likewise, total *Lm* burdens in the intestine and extra-intestinal organs of mice challenged with sessile or planktonic cells of strain F45 are similar to F4244-challenged mice. We did not observe any significant difference in intestinal *Lm* counts for F45 strain; however, significantly (*P* < 0.05) more planktonic cells were found in extra-intestinal organs than the sessile cells (Fig. 5j). In particular, significantly (*P* < 0.05) more planktonic F45 than biofilm-isolated bacteria were detected in the cecum, but not in other sections of the intestine (jejunum, ileum, and colon) (Fig. 5a–e). Whereas in the extra-intestinal organs, infection by planktonic F45 resulted in significantly (*P* < 0.05) more *Lm* counts in MLN and spleen than in the liver (Fig. 5e-g). Furthermore, the presence of biofilm-isolated *Lm* in MLN of three mice and spleen of one mouse could not be detected even after culture enrichment followed by qPCR (Fig. 5e, g). Comparing the overall *Lm* burden in the intestine or extra-intestinal organs between F4244 (clinical isolate) and F45 (food isolate) indicates that F4244 had 1–2 log higher counts, hence it is more invasive than F45 in a mouse model of infection (Fig. 5i, j). These data further reveal that while biofilm-isolated cells are in the process of translocating through the intestinal tissues, planktonic *Lm* cells have already disseminated to the extra-intestinal sites at 24 hpi.

At 48 hpi, F4244 cell burden in both intestinal and extra-intestinal tissues for both sessile cell- and planktonic cell-challenged mice were alike (Fig. 5a, b, d, e, f, h) except for the cecum (Fig. 5c) and spleen (Fig. 5g) where planktonic counts were significantly (*P* < 0.05) higher than the sessile cells. Comparing the total bacterial burden in the whole intestine and extra-intestinal tissues, no significant difference in total bacterial burden in extra-intestinal tissues was observed between planktonic or sessile bacteria-challenged mice (Fig. 5i). Likewise, in F45 infected mice, the burden of planktonic or sessile cells had no significant difference in all intestinal or extra-intestinal organs examined at 48 hpi (Fig. 5a–j).

Collectively these data demonstrate that the biofilm-isolated *Lm* has temporarily attenuated capacity to translocate across the gut barrier and/or to disseminate in the blood/lymphatic circulation during the early phase of infection (12–24 h), while both planktonic and biofilm-isolated *Lm* were able to disseminate to extra-intestinal tissues similarly at 48 hpi.

### Histopathology shows the increased inflammatory response for planktonic cells than the sessile cells

**Fig. 6:**
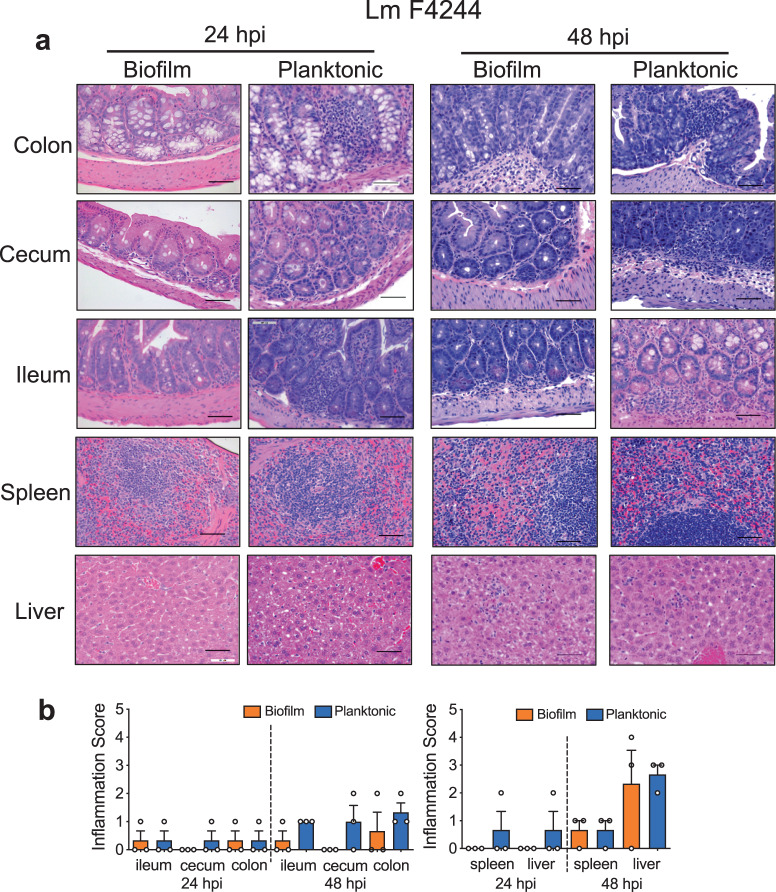
Histopathology analysis of mouse tissues for inflammation. Representative images of hematoxylin and eosin-stained tissue sections of mice challenged with 1 × 10^9^ CFU of F4244 sessile (B) or planktonic (P) cells at 24 and 48 hpi (**a**) and a graph representing histopathological inflammation scores at 24 hpi (**b**, left panel) and 48 hpi (**b**, right panel). Scale bars represent 50 μm.

At 24 hpi, histopathological analysis of planktonic F4244 infected intestinal tissues revealed more polymorphonuclear and mononuclear cells infiltrating villi in mice than the sessile bacteria-infected tissues (Fig. 6a). At the same time, an increased amount of single-cell necrosis and higher inflammation scores were observed in the liver and spleen of planktonic F4244-challenged mice, suggesting planktonic bacteria caused more inflammatory lesions in extra-intestinal organs than the sessile bacteria at 24 hpi (Fig. 6a, b). At 48 hpi, a similar inflammatory lesion was observed in both intestinal and extra-intestinal organs of mice challenged with either planktonic or sessile cells of F4244 (Fig. 6a). The sessile and planktonic cells of the F45 strain also showed similar results as F4244 but the overall inflammatory response was much lower than F4244 (Supplementary Fig. 6). Overall inflammation scores showed that sessile bacteria caused much more lesions in the spleen and liver at 48 hpi compared to 24 hpi, which is consistent with the increased bacterial burdens in these organs (Fig. 6).

### Sessile and planktonic *Lm* with murinized internalin A (InlA^m^) showed similar pathogenicity and systemic dissemination as the wild-type strain

**Fig. 7:**
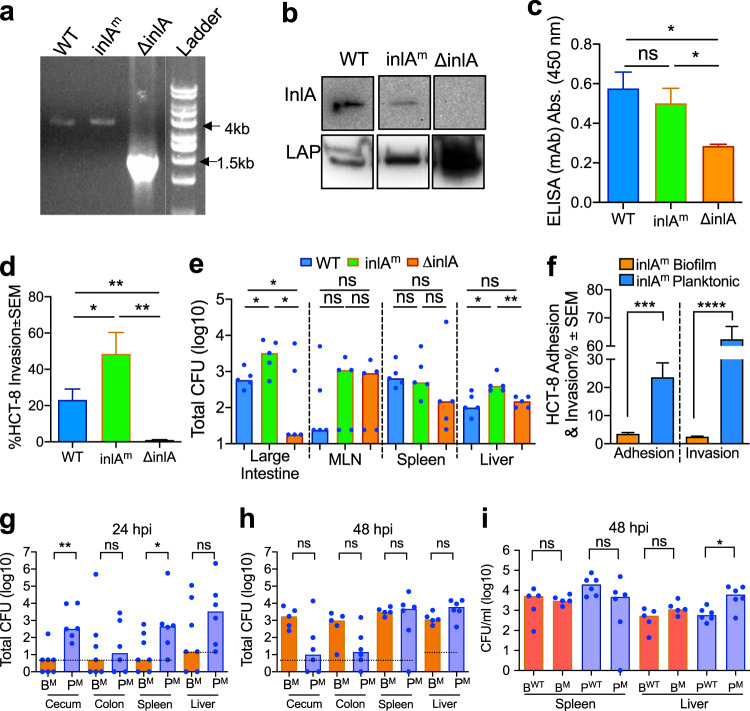
In mouse bioassay, biofilm-isolated and planktonic *L. monocytogenes* with murinized InlA (InlA^m^) display differential tissue distribution. **a** PCR confirmation of the insertion of *inlA*^*m*^ gene in the chromosome of *Lm* F4244 ∆*inlA* using primers inlA.up.5 and inlA.down.3 (Supplementary Table 2). WT (F4244) was used as a positive control. **b** Immunoblots showing expression profile of InlA, and LAP in whole-cell extracts of WT, *inlA*^*m*^, and ∆*inlA*. **c** ELISA showing the positive reaction of anti-InlA mAb to whole-cell preparation of WT, *inlA*^*m*^, and reduced reaction with ∆*inlA*. **d** Percent invasion of WT, *inlA*^*m*^, and ∆*inlA* to HCT-8 cells. Bars represent mean, and a pairwise Student’s *t*-test was used for statistical analysis. **e** Lm WT, *inlA*^*m*^ and ∆*inlA* strain burdens in the large intestine, MLN, spleen, and liver of mice (*n* = 5–6) 96 h after oral challenge (5 × 10^9^ CFU/mouse). Mann–Whitney test was used for statistical analysis. **f** Percent adhesion and invasion of biofilm-isolated and planktonic cells of InlA^m^ strain to HCT-8 cells. Bars represent mean, and a pairwise Student’s *t*-test was used for statistical analysis. **g,** **h** *Lm* burdens in tissues of mice (C57BL/6, male and female, 8–10 weeks old) challenged with murinized InlA^m^ (1 × 10^9^ CFU/mouse) strain of biofilm-isolated (B^M^) or planktonic (P^M^) cells at 24 (**g**) or 48 (**h**) hpi. Mice were pretreated with streptomycin (5 mg/ml) in drinking water for 32 h followed by 16 h in antibiotic-free water before the *Lm* challenge. **i** Comparison of tissue (spleen and liver) burden between WT and InlA^m^ strain for biofilm-isolated (B^WT^ vs B^M^) and planktonic (P^WT^ vs P^M^) cells at 48 hpi. Data for WT were taken from Fig. 5. Bars represent median values, and the Mann–Whitney test was used for statistical analysis in **e**, **g**, **h**. ****P < 0.0001; ***P < 0.0005; **P < 0.005; *P < 0.05; ns, no significance.

To verify the role of InlA in *Lm* pathogenesis in biofilm-isolated sessile cells in the mouse model, we created murinized inlA (InlA^m^) in F4244 by substituting two specific amino acids, S192N and Y369S (Fig. 7)^10^. The *inlA*^*m*^ gene sequencing (Supplementary Fig. 7a–d and Fig. 7a), Western blotting (Fig. 7b) and ELISA (Fig. 7c) confirmed the expression of InlA in the InlA^m^ strain. Besides, InlA^m^ strain also showed significantly (*P* < 0.05) higher invasion into intestinal epithelial HCT-8 cells than the WT (F4244) strain (Fig. 7d) consistent with the results reported for Caco-2 cells^10^. In the mouse experiment, InlA^m^ strain also showed significantly (*P* < 0.05) higher invasion of large intestinal tissues and translocation to the liver after 96 hpi compared to the WT strain (Fig. 7e) as observed before^10^.

We then examined adhesion and invasion of planktonic (P^M^) and biofilm-forming sessile cells (B^M^) of InlA^m^ strain in vitro, and the planktonic cells showed significantly higher adhesion and invasion into HCT-8 cells than the sessile cells (Fig. 7f) similar to WT F4244 cells (Fig. 2). Next, aiming to observe increased invasion and *Lm* tissue burdens in the mouse model of infection, we pretreated the mice with streptomycin (5 mg/ml) for 32 h in drinking water^50^ before oral challenge with sessile and planktonic InlA^m^ strains at 1 × 10^9^ CFU/mouse. The sensitivity of InlA^m^ strain to streptomycin was tested before animal administration and was determined to be 2.5 µg/ml (Supplementary Fig. 7e).

At 12 hpi, as before, *Lm* could not be enumerated by the plating method; hence, the tissue samples were tested for the presence or absence of *Lm*. In the intestinal tissue samples, only 11–33% of mice (n = 9) were positive when challenged with sessile cells while 22–67% of mice (*n* = 9) were positive for planktonic cells. In the extra-intestinal tissues, sessile cells were isolated only from the liver of two mice (22%) while all other tissues (MLN & spleen) were negative. In contrast, 22–56% of mice were positive when challenged with planktonic cells (Table 1).

At 24 hpi, planktonic cells showed significantly (*P* < 0.05) higher invasion into the cecum and spleen than the sessile cells. While in the colon and liver there were no differences (Fig. 7g). These data further demonstrate that even though InlA-dependent invasion was restored in the mouse model, the sessile cells still showed delayed invasion and tissue distribution.

At 48 hpi, there was no statistical difference in planktonic and sessile cells of InlA^m^ strain in the mouse intestinal and extra-intestinal tissues (Fig. 7h). We also compared the tissue distribution patterns of sessile and planktonic cells of both WT (data from Fig. 5) and InlA^m^ strain (data from Fig. 7h) at 48 hpi and no significant differences were observed between these two strains in the spleen and liver except for planktonic cells of InlA^m^ strain in the liver which showed higher (*P* < 0.05) invasion (Fig. 7i). Overall these data show a consistent trend in tissue invasion for sessile and planktonic cells of InlA^m^ and the WT strain confirming the attenuation of translocation of biofilm-forming sessile cells during the early stage (12–24 h) of infection.

### LAP and InlA expression were significantly upregulated in planktonic cells than the sessile cells after exposure to simulated gastrointestinal fluids for 13 h

**Fig. 8:**
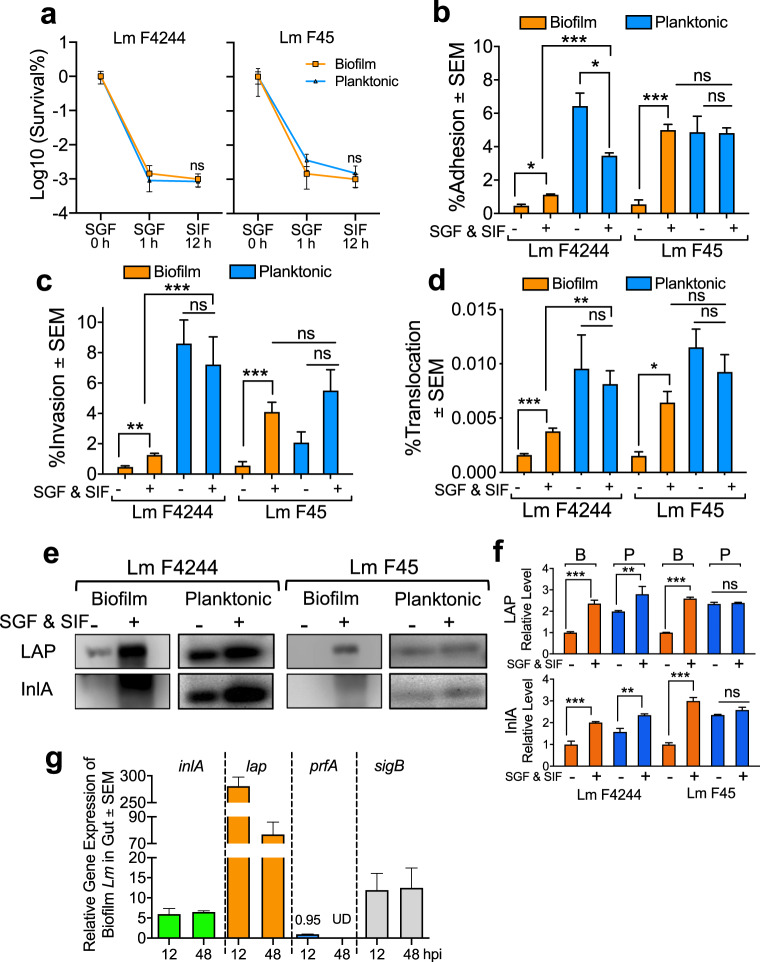
Survival and virulence of biofilm-isolated and planktonic *L. monocytogenes* strain suspended in simulated gastrointestinal fluid. **a** Survival of sessile and planktonic *Lm* F4244 and F45 after sequential exposure to simulated gastric fluid (SGF, pH 2) for 1 h and simulated intestinal fluid (SIF, pH 7) for 12 h. **b**–**d** Comparison of adhesion (**b**), invasion (**c**), and translocation (**d**) rates on Caco-2 cells of SGF (pH 3) and SIF (pH 7)-treated biofilm-isolated and planktonic *Lm* F4244 and F45. **e**, **f** Immunoblot showing LAP and InlA expression in sessile and planktonic cells after exposure to SGF (pH 3) and SIF (pH 7). Immunoblots are representative of three independent experiments. **g** Relative mRNA expression of virulence genes (*inlA* and *lap*) and virulence regulators (*prfA* and *sigB*) in biofilm-isolated InlA^m^ from mice intestinal chymus at 12 or 48 hpi and the same cells before infection using RT-PCR. UD, Undetectable. A pairwise Student’s *t*-test was used for statistical analysis. ***P < 0.0005; **P < 0.005; *P < 0.05.

In vivo data revealed late dissemination of sessile cells to extra-intestinal tissues; therefore, we hypothesized that the sessile cells are either more susceptible to intestinal conditions than that of the planktonic cells or the intestinal condition may suppress the expression of key virulence factors in sessile cells. To verify the first event, we tested the survivability of both biofilm-isolated and planktonic *Lm* cells exposed to simulated gastric fluid (SGF) and simulated intestinal fluid (SIF). Both sessile and planktonic cell viability was decreased by about three logs after 60 min of exposure to SGF and there was no significant difference in cell viability between the two (Fig. 8a). We then examined the adhesion, invasion, and translocation properties of these bacterial cells through Caco-2 cells and analyzed the expression of LAP and InlA proteins. Interestingly, SGF and SIF-exposed sessile cells of both F4244 and F45 strains showed significantly increased adhesion, invasion, and transepithelial translocation through Caco-2 cells compared to the sessile cells that are not exposed to simulated gastrointestinal fluids (Fig. 8b–d). These results further indicate that exposure to gastrointestinal conditions increased virulence attributes in *Lm* sessile cells. In contrast, planktonic cells showed mixed results showing slightly decreased or the same levels of adhesion, invasion, and translocation with or without exposure to SGF and SIF (Fig. 8b–d). Overall, planktonic cells displayed significantly higher adhesion, invasion, and translocation than that of the sessile cells, thus supporting the hypothesis that virulence of planktonic bacteria is significantly higher in the gastrointestinal environment than the sessile bacteria at the very early (12–24 h) stage of infection (Fig. 8c, d).

Immunoblot analysis confirmed a significant increase (2–3-fold) in the expression of LAP and InlA in sessile cells in both F4244 and F45 strains after exposure to SGF and SIF (Fig. 8e, f). Furthermore, significantly increased expression of these proteins was also observed in planktonic cells of the F4244 strain but not in the F45 strain. Overall, the expression of these proteins was significantly higher in planktonic cells than in the sessile cells in F4244 strains (Fig. 8f). Taken together, these data show that overall reduced expression of LAP and InlA in sessile cells relative to the protein expression by planktonic cells may be responsible for decreased adhesion, invasion, and transepithelial migration during the very early stage (12–24 h) of infection.

To validate the hypothesis that sessile cells upregulate virulence genes after oral infection, we quantified the transcriptional expression of virulence genes in sessile InlA^m^ cells from mice intestinal chymus 12 and 48 hpi and compared them with the expression in the same cells before infection. We observed a fivefold higher *inlA* expression in InlA^m^ at both 12 and 48 hpi compared to that of control (InlA^m^ cells before infection) (Fig. 8g). Interestingly *lap* expression was 250- and 70-fold higher at 12 and 48 hpi, respectively (Fig. 8g). The *sigB* expression was 10-fold higher at 12 hpi and maintained at a similar level at 48 hpi (Fig. 8g). In contrast, *prfA* expression remained unchanged at 12 hpi, and it was below the detection limit at 48 hpi (Fig. 8g). These data indicate that the delayed invasion of sessile cells in mice at 24 hpi was possibly because of their lower expression of virulence factors, LAP and InlA, than their planktonic counterparts. Besides, the intestinal environment positively upregulates *lap* and *inlA* expression in sessile cells in the intestine, which could allow the sessile cells to be as invasive as planktonic cells at 48 hpi.

## Discussion

The biofilm-forming ability gives *Lm* the advantage of persistence even for many years on various surfaces in a food processing/production environment, which presumably serves as a primary source for food contamination^19–22^. *Lm* has been routinely isolated from meat and dairy^51^ processing plants. The persistence of pathogens on the abiotic surface is facilitated by their ability to form a biofilm, in which cells experience a wide range of stress thus show physiological and genetic heterogeneity allowing them to be more resistant to antimicrobials, and to survive in limited nutrient and oxygen tensions^27,52,53^. Although several studies have reported reduced expression of virulence genes in sessile cells, the pathogenic potential of these cells has not been tested using either in vitro or in vivo models. Therefore, we studied the virulence of biofilm-isolated sessile cells of *Lm* using both cell culture and animal models, and the expression of virulence genes to support the observed phenotype, especially between 12 and 48 hpi.

The biofilm-forming capability of over 100 *Lm* strains was screened and all formed biofilms of varying degrees on a polystyrene surface, and food isolates, in general, had significantly higher biofilm-forming capacity than the clinical isolates (Fig. 1). These observations agree with the previous studies^27,54,55^. Among the different serotypes examined, isolates of serovar ½a and ½c (Lineage II) are stronger biofilm formers than the isolates of ½b and 4b (Lineage I), which are in accordance with another study^55^. We also observed that many strains were weak biofilm-former and their persistence on surfaces may be doubtful; however, studies have demonstrated that mixed-species biofilms possibly facilitate the persistence of such weak biofilm-forming pathogens^56,57^.

Biofilm-forming cells experience stress and exhibit physiological and genetic heterogeneity^52^; thus, we were curious about their dynamics of infectivity in cell culture and mouse models. Five *Lm* strains of food and clinical origins representing the major outbreak causing serotypes with diverse biofilm-forming phenotypes were selected for in vitro cell culture experiments. All biofilm-isolated cells we tested irrespective of food or clinical origins were less adhesive, invasive, and cytotoxic and showed reduced ability to traverse across the Caco-2 epithelial barrier than the planktonic cells, suggesting these cells are less virulent compared to the planktonic cells (Figs 2 and 3). We analyzed the expression of key virulence proteins (LAP, InlA, and LLO) that are responsible for *Lm* invasion, paracellular translocation, and intracellular persistence. We observed significantly reduced expression of these proteins in sessile cells at the transcriptional and translational levels (Figs 3 and 4), which may explain the reason for reduced virulence of sessile cells in in vitro cell culture experiment. However, the contribution of other virulence factors including ActA cannot be ignored. ActA, a PrfA, and SigB regulated protein known to contribute to biofilm formation and intestinal colonization^58,59^ may also be affected in sessile cells for the delayed invasion and tissue distribution in mice. Furthermore, reduced *inlA* expression in sessile cells is in agreement with others who also observed similar reduced InlA expression in biofilm-isolated cells^36,37^. Besides, mRNA of gene *sigB*, coding a stress response regulator, was also downregulated to around 25% in sessile cells of both *Lm* strains compared to their planktonic counterparts (Fig. 4). SigB has been implicated in *Lm* biofilm formation^39^ and it also regulates InlA expression^60^. The observed suppression of SigB and consequent InlA expression in sessile cells possibly is responsible for reduced *Lm* adhesion and invasion into the intestinal epithelial cells, which was further supported by a proteomic analysis that indicated downregulation of SigB-regulated proteins^36^.

Interestingly, *prfA* mRNA in *Lm* F45, a strong biofilm-former, was expressed at a similar level for both sessile and in planktonic cells; however, its level was downregulated by 25% in *Lm* F4244 (a moderate biofilm-former) sessile cells than that of the planktonic cells (Fig. 4). These observations differ from a previous study where PrfA is reported to positively regulate biofilm formation^61^ and a strain (*Lm*10403S) overexpressing *prfA* showed higher biofilm-forming ability than the WT. Our data further imply that PrfA-regulated biofilm formation may vary from strain to strain which requires further investigation. Although PrfA is a key regulator for the expression of multiple virulence factors including InlA^62^, our qRT-PCR results further suggest that decreased expression of *inlA* in sessile cells is not always coupled with decreased expression of *prfA* (Figs 3 and 4) since InlA can be expressed independently of PrfA regulation^63^.

To confirm in vitro cell culture results in a mouse model, we challenged mice orally with 48-h-old sessile cells or 24-h-old planktonic cells of moderate biofilm-forming clinical strain (F4244) and a strong biofilm-forming food isolated strain (F45). At 12–24 hpi in mice, sessile cell burden in intestinal and extra-intestinal tissues was undetectable or very low while the planktonic burden was significantly high and infectivity was comparable to the in vitro cell culture data indicating sessile cells are less invasive. However, at 48 hpi, burdens of both sessile and planktonic cells in mouse tissues were comparable, suggesting that sessile cells are equally invasive as planktonic cells after the early stage (12–24 hpi) of the gastrointestinal phase of infection; however, the rate of bacterial tissue distribution and disease progression was variable (Figs 5 and 6). To explain such discrepancy in intestinal epithelial cell invasion in the early stage (12–24 hpi) of infection, we hypothesized that possibly sessile cells are highly susceptible to antimicrobials present in intestinal fluids or expression of adhesion and invasion-related proteins are suppressed in intestinal fluids. Therefore, we examined survival and protein expression in sessile cells suspended in SGF (pH 3) and SIF (pH 7.0) that contain HCl, enzymes, and bile salts^64^. In SGF + SIF, we did not observe any significant difference in *Lm* viability between sessile and planktonic cells but observed differential expressions of LAP and InlA, the two key virulence factors that are responsible for *Lm* translocation across the gut epithelial barrier^3,4,9^. Though the expression of both LAP and InlA were significantly upregulated in sessile cells in SGF and SIF, overall expression in planktonic cells was significantly higher than the sessile cells (Fig. 8e). These findings suggest that the gastrointestinal environment may help the sessile cells to quickly transition to a fully virulent state and may also explain the observed similar intestinal and extra-intestinal tissue burdens for both biofilm and planktonic cells at 48 hpi.

Our hypothesis is also supported by the observation that *inlA* and *lap* mRNA in sessile cells were upregulated after they arrive in the mouse intestine for 12 h (Fig. 8g). However, the expression of *inlA* mRNA maintained at a similar level and the expression of *lap* even decreased at 48 hpi (Fig. 8g), suggesting the expression of the virulence genes may not continue to increase with increasing residence time in the intestine. During this period, expression of regulatory genes, *prfA*, was unaffected while the *sigB* level increased several-fold consistent with a previous report, which showed SigB-mediated upregulation of several virulence genes, including *inlA*, is critical for *Lm* to switch global transcription from saprophytism to virulence while residing in the intestine^65^. This study further reinforces the importance of *sigB* in virulence gene expression in sessile cells during the intestinal phase of infection. Although the gastrointestinal environment is known to upregulate both LAP^66–68^ and InlA expression^36,37,69^, here, we provide evidence for the expression of these two proteins in biofilm-isolated cells in the mouse intestine.

In mice, InlA-mediated transcytosis is absent due to a lack of interaction between InlA and its cognate receptor, E-cadherin^70^; thus, LAP-mediated *Lm* translocation is considered the predominant gut-barrier crossing mechanism in mice during the early (12–24 h) stage of infection^3,4,8^. Hence, the observed reduction in LAP expression in sessile cells is considered a major contributory factor towards impaired *Lm* translocation in the intestinal and extra-intestinal tissues early in the infection process (12–24 h) (Fig. 5).

To further investigate the role of InlA in sessile cell infection in the mouse model, we generated InlA^m^ strain^10^ and the intestinal and extra-intestinal tissue distribution of sessile and planktonic cells of InlA^m^ surprisingly followed the same trend as the WT strain at 12, 24, and 48 hpi (Table 1 and Fig. 7). InlA^m^ strain still did not show increased tissue distribution of either sessile or planktonic cells at 48 hpi compared to the WT. This result was expected since previous studies have shown that differential tissue distribution of InlA^m^ and WT strains occur only after 72–96 h infection in mice^10,71^ and this was again verified in our study (Fig. 7e).

Furthermore, this experiment was conducted with mice that were even pretreated with streptomycin for 32 h in the drinking water to disrupt resident microflora^2^ and we still did not observe increased tissue distribution of either sessile or planktonic cells of InlA^m^ strain at 48 hpi compared to the WT (Fig. 7i). The failure to observe increased tissue distribution is believed to be due to increased sensitivity of F4244 strain to streptomycin (MIC, 2.5 µg/ml) (Supplementary Fig. 7e) used in the drinking water, thus possibly affected its survival and tissue distribution. On the other hand, the previous study^2^ used the 10403S strain which is highly resistant to streptomycin (~1 mg/ml) (Supplementary Fig. 7e), thus ensuring its survival and increased tissue dissemination (2–4 log) in the animal pretreated with streptomycin. To study bacterial invasiveness in an antibiotic-pretreated animal model, it is imperative to use a pathogen that is resistant to the same antibiotic used for microbiota disruption. For example, van der Waaij et al.^72^ demonstrated that the dissemination and persistence of infectious *E. coli* in mice was facilitated only when the bacterium is resistant to the pre-exposed antibiotics. Similarly, Hentges et al.^73^ reported that the burdens of clindamycin-sensitive *Pseudomonas aeruginosa* in MLN and liver of clindamycin-treated mice were lower than the burdens in untreated mice. Further experiments may be necessary to validate the antibiotic effect of our *Lm* strain (F4244) invasion by using a streptomycin-resistant strain which we plan to investigate in the future.

LLO is an important virulence factor required for *Lm* persistence during intracellular lifestyle^74^ and is also responsible for epithelial and lymphocyte apoptosis^46,47,49,75^. In addition, LLO has been implicated to aid *Lm* dissemination from the gastrointestinal tract to extra-intestinal tissues^76^. In this study, we observed reduced LLO expression in sessile cells (Figs. 3 and 4), which agrees with one study^36^, but contradicts with other^77^, where researchers report that biofilm formation does not affect bacterial ability to produce LLO. Interestingly, in another pathogen (*Bacillus cereus*), researchers^77^ observed reduced expression of Hemolysin BL and other enterotoxins (CytK and EntC) in biofilm cells and consequently reduced cytotoxicity on both HeLa and MDA cells^78^. Collectively, these data imply that impaired toxin synthesis in biofilm cells affects bacterial virulence.

In summary, our data indicate that sessile cells are less invasive in cultured cell lines and during the early stage (12–24 h) of infection in an animal model possibly due to reduced expression of regulatory proteins (PrfA and SigB) and virulence factors (LAP, InlA, and LLO). However, both sessile and the planktonic cells showed similar extra-intestinal tissue burdens at 48 hpi and sessile cells are equally infective as planktonic cells but the dynamics of infection may vary between sessile and planktonic cells with possible differential disease onset or incubation period. Furthermore, in vitro cell culture experiment routinely used for virulence potential determination is found to be unreliable for assessing the pathogenic potential of biofilm-forming cells because it measures the pathogenic event over a short period (1–2 h). On the other hand, an animal model provides comprehensive pathogenic events over a prolonged period in physiologically relevant conditions and, thus, is most reliable for studying the pathogenesis of biofilm-isolated cells.

## Methods

### Bacterial strains

Food (64) and clinical (46) isolates and several mutant *Lm* strains were used in this study (Supplementary Table 1). Cultures were stored in brain heart infusion broth (Acumedia) with 25% glycerol at −80 °C. To revive cells, the frozen stock cultures were first streaked on a tryptic soy agar plate containing 0.6% yeast extract (TSAYE; Becton Dickinson, Franklin Lakes, NJ), and incubated at 37 °C overnight. Then a single colony was inoculated into 4 ml tryptic soy broth supplemented with 0.6% yeast extract (TSBYE), which was further incubated at 37 °C for 16–18 h to obtain fresh cultures. The cultures of *Lm* 10403 S Δ*prfA* and F4244 Δ*inlA* were prepared in the same way, while *lap͞* strain in a medium containing erythromycin (10 µg/ml)^79^ (Supplementary Table 4).

### Development of *Lm* F4244 expressing murinized Internalin A (InlA^m^)

Murinization of InlA in F4244 (4b) was accomplished by following a method described before^10^ and outlined in Supplementary Fig 7. Briefly, the sequence between nucleotide 494 and 1485 of the *inlA* gene in *Lm* F4244 (GenBank: CP015508.1)^80^ including mutations for the two amino acids substitution was synthesized by GenScript and amplified using Q5 high-fidelity polymerase (New England BioLabs). The other two fragments, upstream 800 base pairs to *inlA* nt 493 and *inlA* nt 1486 to downstream 800 base pairs, were individually amplified using *Lm* F4244 gDNA as the template. Then, a mixture of the three segments was used as the templates, amplified, and combined into a whole fragment by PCR. The whole fragment and a temperature-sensitive suicide plasmid, pHoss1 (Addgene), were digested by *Nco*I and *Sal*I and ligated using T4 ligase (New England BioLabs). Ligated pHoss1::*inlA*^*m*^ was transformed and maintained in *E. coli* DH5α (Invitrogen). Purified pHoss1::*inlA*^*m*^ was electroporated into electrocompetent *Lm* F4244 *∆inlA* at 2000 V (BTX Electroporation System), and transformants were selected on BHI agar plates supplemented with 10 μg/ml erythromycin^81^. The *inlA*^*m*^ knock-in mutant strain was selected as before^82^ and the specific mutation in *inlA*^*m*^ ORF was confirmed by Sanger sequencing (Eurofin) using four primers of both directions (Supplementary Fig. 7). Colony PCR to screen the knock-in mutant was conducted using the Platinum II Hot-Start PCR Master Mix (Invitrogen). The expression of InlA^m^ in the whole-cell extract was verified in western blot by using anti-InlA mAb-2D12 (ref. ^83^). The surface expression of InlA^m^ was also confirmed by whole-cell ELISA as before^7^.

### Mammalian cells

Caco-2 cell line (ATCC, Manassas, VA) was seeded and incubated in T-25 tissue culture flasks (TPP, Switzerland) with high glucose DMEM (HyClone, Logan, UT) and 10% (vol/vol) fetal bovine serum (D10F; Atlanta Biologicals) at 37 °C with 7% CO_2_, and 95% relative humidity until the confluence was achieved^84^. The cell monolayer in the flask was trypsinized (Hyclone) and about 1–2 × 10^4^ cells were seeded in each well of 24-well tissue culture plates (TPP, Switzerland) or Transwell inserts with 3.0 μm pores, respectively, for 14–21 days until 95% confluence and polarization were achieved^3,84^. HCT-8 (ATCC) human ileocecal cell line was prepared with the same procedures with minor adjustments. Cells (4.5 × 10^4^/500 μl) were seeded in each well of a 48-well tissue culture plate (TPP, Switzerland) and incubated for 4–5 days^6^. Ped-2E9 cell line, a B cell hybridoma^44^, was cultured in the same conditions and incubated in the 75 cm^2^ flask (TPP, Switzerland) for 3–4 days before being used for experiments^44,47^.

### Biofilm assay and the preparation of biofilm and planktonic culture

The microtiter plate biofilm assay^41^ was followed to quantify biofilm formation with slight modification. Optical density of overnight grown *Lm* TSBYE culture was measured at 595 nm and standardized to 1.2. Then, the culture was diluted with Modified Welshimer’s Medium (MWB, Himedia) by 1 to 40 and 150 µl MWB was aliquoted into six wells on a 96-well tissue culture-treated microtiter plate (Corning, NY) and incubated at 30 °C for 48 h^55^. To quantify the formation of biofilm in each well, supernatant media was pipetted out and each well was washed thrice with 10 mM sterile phosphate-buffered saline (PBS) to remove loosely attached cells. After air-drying microtiter plate for 15 min, 150 μl of 0.1% CV solution was added to each well to stain the biofilm cells and incubated for 45 min at room temperature. Further, each well was washed three times with sterile water to remove residual CV stain, and, after air-drying wells for 15 min, an aliquot of 200 μl of 95% ethanol was added into each well and incubated for 15 min under ambient temperature to destain the biofilms. Finally, the ethanol solution from each well was transferred to a fresh flat-bottom microtiter plate to measure absorbance at 595 nm.

To collect sessile bacteria, the MWB culture (40 ml) was prepared in the same way but inoculated into a tissue culture-treated petri dish with 60.1 cm^2^ growth surface area (TPP, Switzerland). After growth at 30 °C, each plate was rinsed with 5 ml sterile PBS twice and 5 ml PBS was added before detaching biofilm by 15 min sonication in a water bath (iSonic, Chicago, IL). Planktonic bacteria were prepared by incubating the MWB culture in test tubes under the same conditions (at 130 r.p.m. for 24 h).

### In vitro bacterial adhesion, invasion, translocation, and cytotoxicity assays

Bacterial adhesion and invasion were examined using Caco-2 (colon) and HCT-8 (ileocecal) cell lines. *Lm* planktonic and biofilm cultures were prepared as described above, washed thrice with sterile PBS, and diluted in D10F^84^ to proper concentrations. Both sessile and planktonic bacteria in D10F were diluted and added to epithelial cell monolayers in a well at a multiplicity of infection (MOI) 10. For adhesion assay, monolayers were washed once after 30 min infection^5^ and bacteria were released from mammalian cells into 500 µl sterile 0.1% Triton X-100, serially diluted, and enumerated by plating on BHI agar plates (BHIA). For invasion assay, cells were incubated for 2 h^85^, washed, and then monolayers were incubated with D10F containing 50 µg/ml gentamicin to eliminate extracellular bacteria before lysing mammalian cells by 0.1% Triton X-100. The amount of invaded *Lm* was enumerated by plating on BHIA. Adhesion and invasion rates were calculated by dividing bacterial cell numbers from lysed cells by the number of inoculums.

Translocation assay was performed using Caco-2 cells seeded in a Transwell setup for 14–21 days as described before^3,84^. Briefly, washed bacterial cells were added to the apical well at an MOI 100 and incubated at 37 °C for 4 h in a CO_2_ incubator. The liquid (500-µl aliquot) from the basal well was removed, diluted, and plated on BHIA for enumeration. The transepithelial electrical resistance (TEER) values of each well were measured before and after translocation experiments to monitor the integrity of cell monolayers. The wells with TEER values between 400 and 600 (﻿200 Ω/cm^2^) were used for experiments.

For cytotoxicity assay, freshly grown Ped-2E9 cells were counted by Trypan blue staining and resuspended in 500 µl D10F with the final cell concentration of approx. 10^6^ cell/ml. Bacteria were added to achieve an MOI 10 and incubated at 37 °C for 2 h^48^. Ped-E9 cells were stained with Annexin V-PE and 7-AAD (BD, Franklin Lakes, NJ) following the vendor’s protocol. The Ped-2E9 cells which were viable, in the early apoptosis phase or dead were recognized as both Annexin V and 7-AAD negative, Annexin V positive and 7-AAD negative, and both positive Annexin V and 7-AAD positive, respectively. Labeled cells were analyzed by BD Accuri™ C6 that detects Annexin V-PE in FL-2 and 7-AAD in FL-3, and at least 10,000 events were collected from each sample. As blank controls, two samples of Ped-2E9 cells went through all the labeling procedures the same as the testing samples but without bacterial infection. The percentage of Annexin V-positive events of each sample was calculated by subtracting the average percentage of Annexin V-positive events in blank controls from the same experiment. To confirm proper labeling, bacteria-treated Ped-2E9 cells were also examined under a fluorescence microscope (Leica, Buffalo Grove, IL) after staining with Annexin V-FITC and 7-AAD, respectively^48,86^.

Cytotoxicity was also tested on a Caco-2 cell line by assessing intracellular LDH release^87^. Caco-2 cells in 24-well plates were incubated with sessile or planktonic *Lm* at MOI 10 for 2 h at 37 °C. Released LDH in the supernatant was quantified using Pierce LDH Cytotoxicity Kit (Thermo Scientific, Frederick, MD). Supernatant from non-treated cell monolayers and cells lysed with 0.1% Triton X-100 were used as negative and positive controls to determine 0 and 100% cytotoxicity, respectively.

### Protein extraction and Western blotting

The whole-cell protein, secreted protein, cell wall protein, and intracellular proteins were extracted and analyzed separately using Western blot to compare the expression of key virulence genes in biofilm-isolated and planktonic *Lm*^66^.

Briefly, to extract whole-cell protein, approximately 1 × 10^8^ to 1 × 10^9^ biofilm-isolated or planktonic *Lm* cells were harvested, washed, and resuspended into 100 μl PBS. Then, the bacterial cultures were kept in ice and sonicated for three 15 s cycles. After centrifugation (14,000*g* for 10 min at 4 °C), whole-cell protein in the supernatant was collected. Protein from the cell wall, intracellular, and supernatant fractions were also extracted^66^ and quantified using BCA (Thermo Scientific) to standardize loading amount. To collect supernatant protein secreted by *Lm* in biofilm, biofilm was incubated in tissue culture-treated Petri dish (TPP) for 24 h, rinsed to remove loosely attached bacteria and added with the same volume of fresh MWB medium for another 16 h at 30 °C. Secreted protein in the supernatant from biofilm cells was extracted from the MWB medium after 48 h while from planktonic cells after 24 h at 30 °C. After standardizing the loading amount, quality, and quantity of protein samples were analyzed using sodium dodecyl sulfate-polyacrylamide gel electrophoresis (SDS-PAGE; 10% acrylamide). Proteins were transferred to a hydrophobic membrane (PVDF) and immunoprobed with antibodies to LAP, and InlA (all from our lab, Supplementary Table 4) and LLO (Cat # ab200538; Abcam, Cambridge, UK). All blots/gels derive from the same experiment and were processed in parallel. See Supplementary Information for original blots.

### RNA extraction and quantitative reverse transcription PCR

Isolation and quantification of mRNA from biofilm-isolated *Lm* from the intestinal chymus were performed as described before^88^ with some modifications. Chymus from the ileum, cecum, and colon were collected into a 2 ml screw-cap tube (BioSpec) pre-filled with 1 ml cold PBS and 2 glass beads (5 mm diameter) (BioSpec), and homogenized using FastPrep 5 G (2 cycles of 6 m/s treatment for 40 s; MP Biomedicals) to release bacteria. The homogenate was then combined into 9 ml of cold PBS and centrifuged (250*g*, 5 min) and the supernatant containing bacteria was filtered through a sterile cell strainer (mesh size 40 μm; Fisher), diluted, and plated on MOX to quantify *Lm*. The filtrate was centrifuged (8000*g*, for 5 min at 4 °C), rinsed twice with cold PBS, resuspended into 1 ml TRIzol containing about 0.3 mg glass beads (0.1 mm diameter; BioSpec), and stored in a 2 ml screw-cap tube (BioSpec) at −80 °C. RNA was extracted from bacteria by one cycle of 6 m/s treatment for 40 sec using FastPrep 5G. After centrifugation (8000g, 10 min at 4 °C) RNA in supernatant was purified using Direct-zol™ RNA Miniprep Plus kit (Zymo Research), treated with DNase, and purity was assessed using NanoDrop™ 2000 (Thermo Fisher Scientific). cDNA was synthesized using RNA (2–500 ng, *A*_260_/*A*_280_~2.0, *A*_260_/*A*_230_ > 2.0) and Maxima H Minus Reverse Transcriptase (Thermo Scientific) and random hexamer primers. To quantify the copy number of target genes in cDNA, we first constructed standard curves of gene copy numbers and Ct values. DNA with known copy numbers of each gene was prepared by PCR (Supplementary Table 2 and Supplementary Fig 4), purified, quantified using NanoDrop, serially diluted (about 10–10^9^ copy/μl) and used as templates for quantitative PCR (qPCR) using PowerUp SYBR^®^ Green Master Mix (Applied Biosystems) and qPCR-specific primers (Supplementary Table 2) in a StepOnePlus™ Real-Time PCR System (Applied Biosystems). The qPCR primers were all self-designed, and their potential secondary structures were predicted using the OligoAnalyzer Tool (IDT). The relative expressions of virulence (*inlA* and *lap*) and regulator (*prfA* and *sigB*) genes in biofilm-isolated *Lm* from intestinal chymus 12 or 48 hpi were compared with those expressed in the same culture before oral infection using the following equation:

1 $${\mathrm{relative}}\,{\mathrm{expression}} = \frac{{{\mathrm{total}}\,{\mathrm{copy}}\# \,{\mathrm{of}}\,{\mathrm{target}}\,{\mathrm{genes}}\,{\mathrm{in}}\,{\mathrm{chymus}}(12\,{\mathrm{or}}\,48\,{\mathrm{hpi}})}}{{{\mathrm{CFU}}\,{\mathrm{of}}\,{\mathrm{Lm}}\,{\mathrm{in}}\,{\mathrm{chymus}}(12\,{\mathrm{or}}\,48\,{\mathrm{hpi}})}} \\\times \frac{{{\mathrm{CFU}}\,{\mathrm{of}}\,{\mathrm{biofilm}}}}{{{\mathrm{total}}\,{\mathrm{copy}}\# \,{\mathrm{of}}\,{\mathrm{the}}\,{\mathrm{target}}\,{\mathrm{gene}}\,{\mathrm{in}}\,{\mathrm{biofilm}}}}$$

where “biofilm” denotes biofilm-isolated *Lm* before infection.

To compare virulence gene expression between biofilm-isolated and planktonic *Lm* from non-intestinal samples, cultures were prepared as described above and approximately 10^8^ CFU was collected after washing twice with PBS (8000*g* for 3 min at 4 °C) and resuspended in 1 ml of TRIzol® reagent. Then, RNA extraction and cDNA quantification were carried out as above. The relative expression of *lap, inlA, prfA*, and *sigB* in biofilm-isolated cells compared to planktonic cells were calculated using the following equation:

2 $${\mathrm{Relative}}\,{\mathrm{expression}} = \frac{{{\mathrm{total}}\,{\mathrm{copy}}\# {\mathrm{of}}\,{\mathrm{target}}\,{\mathrm{gene}}\,{\mathrm{in}}\,{\mathrm{biofilm}}}}{{{\mathrm{CFU}}\,{\mathrm{of}}\,{\mathrm{biofilm}}}} \\\times \frac{{{\mathrm{CFU}}\,{\mathrm{of}}\,{\mathrm{planktonic}}}}{{{\mathrm{total}}\,{\mathrm{copy}}\# \,{\mathrm{of}}\,{\mathrm{the}}\,{\mathrm{target}}\,{\mathrm{gene}}\,{\mathrm{in}}\,{\mathrm{planktonic}}}}$$

where, “biofilm” and “planktonic” denote biofilm-isolated and planktonic cells.

### Mouse pathogenicity assay

The animal experiment protocol (No. 1201000595) was approved by the Purdue University Animal Care and Use Committee. C57BL/6 male and female mice (8–10 weeks old) from our breeding colony were used. To characterize the in vivo pathogenicity of InlA^m^ strain, mice (*n* = 5–6/treatment) were orally gavaged with 100 µl of freshly prepared cultures (5 × 10^9^ CFU in PBS) of *Lm* F4244 WT, InlA^m^, or ∆inlA using a stainless steel ball-end needle (Popper)^3^ and sacrificed 96 hpi.

To determine the pathogenicity of sessile and planktonic *Lm* strains, F4244 (WT), InlA^m^ or F45, each mouse was orally challenged with 100 µl of freshly prepared cultures (1 × 10^9^ CFU in PBS/mouse). In a separate experiment, mice were pretreated with streptomycin (5 mg/ml) in the drinking water for 32 h followed by 16 h without antibiotics until bacterial gavage^2,50^. Food was withdrawn 8 h before gavage. Mice (*n* = 4–9/treatment) were sacrificed 12, 24, and 48 h after infection by CO_2_ asphyxiation. Blood was collected through cardiac puncture and 100 μl of blood was mixed with 400 μl 0.4% sodium citrate solution as an anticoagulant and further diluted to enumerate by plating. Extra-intestinal organs were collected and homogenized in 4.5 ml (mesenteric lymph node, spleen, and kidney) or 9 ml (liver) buffered *Listeria* enrichment broth (BLEB: Neogen) supplemented with 0.1% Tween-20 and antimicrobial supplement (Neogen). After sectioning the intestine into jejunum, ileum, cecum, and colon, internal contents were removed and the intestinal sections were split open before washing (2×) and incubation in sterile D10F containing 50 μg/ml gentamicin at ambient temperature for 2 h to eliminate extracellular bacteria. Then, each part of the intestine was individually homogenized in 1 ml BLEB, diluted in PBS, and plated on Modified Oxford plates (MOX; Neogen) to enumerate invaded bacterial load.

Since the viable bacterial counts in most organs from 12 h infected mice were lower than the detection limit by a standard plating method, the presence of *Lm* in these samples was determined by culture enrichment followed by quantitative PCR. Homogenized samples in BLEB were incubated at 37 °C overnight and 100 μl of each culture was plated on MOX plates. Colonies producing black pigment on MOX plates were transferred into 4 ml BHI broth incubated at 37 °C overnight for the final enrichment. DNA was extracted from cultures using the boiling method, and qPCR using SYBR^®^ Green Master Mix (Applied Biosystems) and targeting two specific virulence genes (*prfA* and *inlA*) (Supplementary Table 2) were performed to determine the presence of *Lm*. For the samples that did not form colonies on MOX plates, DNA was directly extracted from enriched BLEB samples before qPCR.

For histopathology analysis, a small section of the spleen, liver, and parts of the intestine was fixed in formalin (10%) and stained with hematoxylin and eosin to be microscopically examined and scored by a board-certified veterinary pathologist as before^3^.

### Bacterial survival and virulence after exposure to simulated gastrointestinal fluids

To assess the survival of biofilm-isolated and planktonic *Lm* in gastrointestinal conditions, SGF and SIF was prepared as before^64^ with an exception. Luria-Bertani (LB) was substituted with distilled water as the solvent for SIF. Biofilm-isolated and planktonic bacteria were rinsed once with sterile deionized (DI) water, resuspended in SGF (pH 2), and incubated at 37 °C with the agitation of 120 r.p.m. for 1 h. Viable bacterial counts were enumerated at 0- and 60-min post-SGF treatment by diluting and plating on BHIA. After SGF treatment, bacterial cultures were rinsed once with sterile DI water, resuspended in SIF (pH 7) for another 12 h incubation at the same conditions, and enumerated after SIF treatment.

Adhesion, invasion, and transepithelial translocation of SGF and SIF-treated or non-treated sessile and planktonic bacteria were tested on Caco-2 cell monolayers as above. At the same time, whole-cell proteins were extracted from treated and non-treated cultures to assess the expression of LAP and InlA using immunoblotting. Instead of SGF (pH 2), bacteria for in vitro virulence tests and immunoblotting were treated with SGF (pH 3) to ensure the availability of an adequate number of viable cells for the experiment. Other conditions remained the same as the survival test.

### Statistical analysis

Experimental data were analyzed using GraphPad Prism (La Jolla, CA) software. *P* values and the type of statistical analysis performed are described in the figure legends. The Mann–Whitney test was used to determine statistical significance for mouse microbial counts and data are presented as median. In other experiments, comparisons between treatment and control were performed using the unpaired Student’s *t*-test, or by one-way or two-way analysis of variance with Tukey’s multiple-comparison test, and data are presented as the mean ± standard error of the mean (SEM).

### Reporting summary

Further information on experimental design is available in the Nature Research Reporting Summary linked to this paper.

## Supplementary information

Supplementary Information

Reporting Summary

## Data Availability

The authors declare that all the data supporting the findings of this study are available within the article and its Supplementary Information file. Data are also available from the corresponding author upon request.
