# Supplementary material for: Biofilm-isolated Listeria monocytogenes exhibits reduced systemic dissemination at the early (12–24 h) stage of infection in a mouse model
Source: NPJ Biofilms Microbiomes. 2021 Feb 8;7:18. doi: 10.1038/s41522-021-00189-5 (PMC7870835; doi:10.1038/s41522-021-00189-5)
Supplement: Supplementary file 1 — Supplementary Information [file 41522_2021_189_MOESM1_ESM.pdf]

Supplemental Information for

Biofilm-isolated *Listeria monocytogenes* exhibits reduced systemic dissemination at the early (12-24 h) stage of infection in a mouse model

Xingjian Bai[1,5], Dongqi Liu[1,5], Luping Xu[1,5], Shivendra Tenguria[1,5], Rishi Drolia[1,5], Nicholas L.F. Gallina[1,5], Abigail D. Cox[2], Ok-Kyung Koo[3,4] and Arun K. Bhunia[1,2,5*]

[1]*Molecular Food Microbiology Laboratory, Department of Food Science, Purdue University, West Lafayette, IN, United States of America.*

[2]*Department of Comparative Pathobiology, Purdue University, West Lafayette, IN, United States of America.*

[3]*Department of Food and Nutrition, Gyeongsang National University, Jinju, Republic of Korea*

[4]*Institute of Agriculture and Life Science, Gyeongsang National University, Jinju, Republic of Korea*

[5]*Purdue Institute of Inflammation, Immunology and Infectious Disease, Purdue University, West Lafayette, IN, United States of America.*

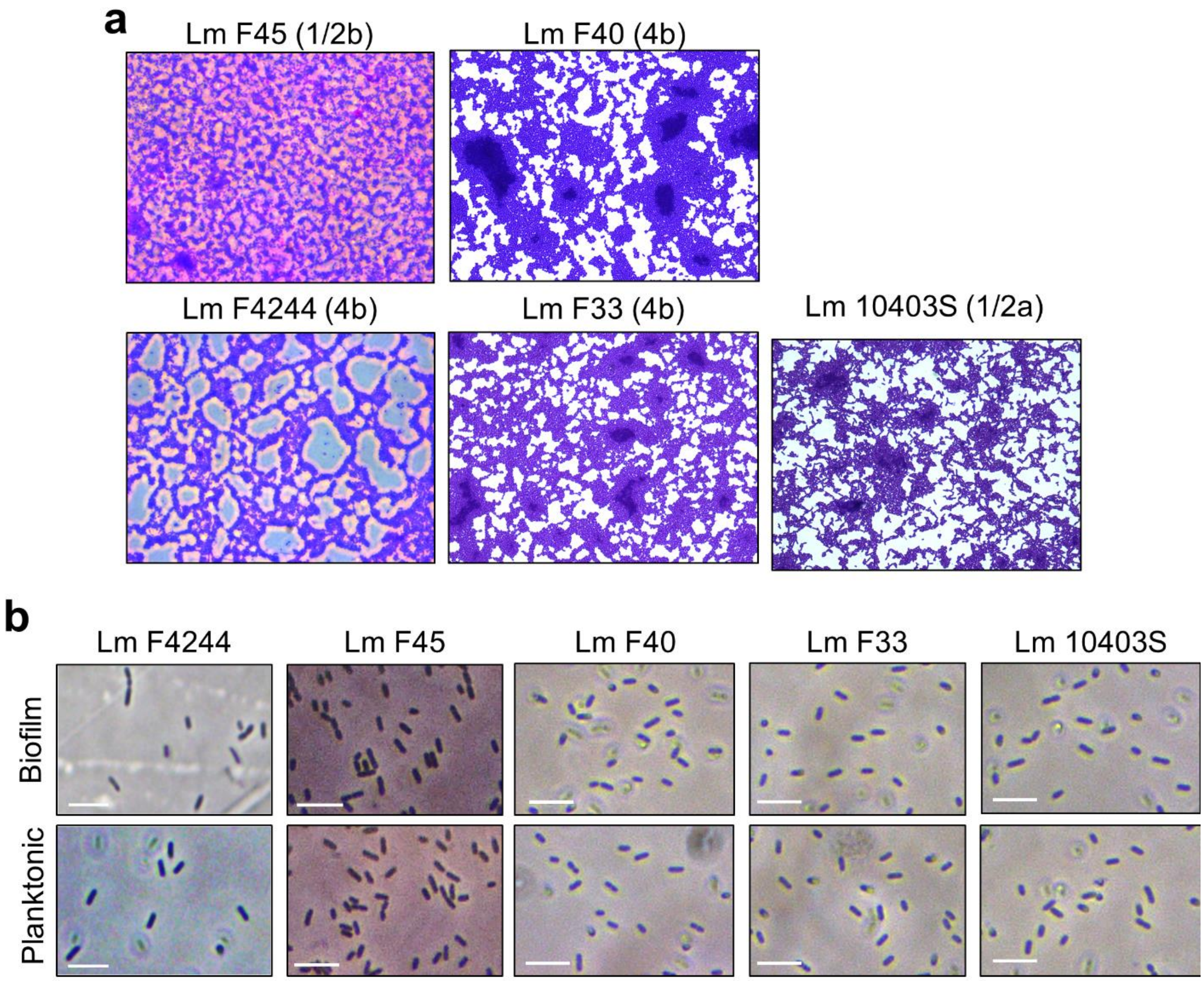

Supplementary Fig. 1. (**a**) Visualization of biofilm formation by high (*Lm* F45 and F40) and moderate (F4244, F33, and 10403S) biofilm former strains of *L. monocytogenes* isolates on glass slides after crystal violet staining. (**b**) Morphological comparison of biofilm-isolated and planktonic *Lm* F4244, F45, F40, F33, and 10403S cells using phase-contrast microscopy shows o significant difference in cell length. Scale bars represent 5 μm.

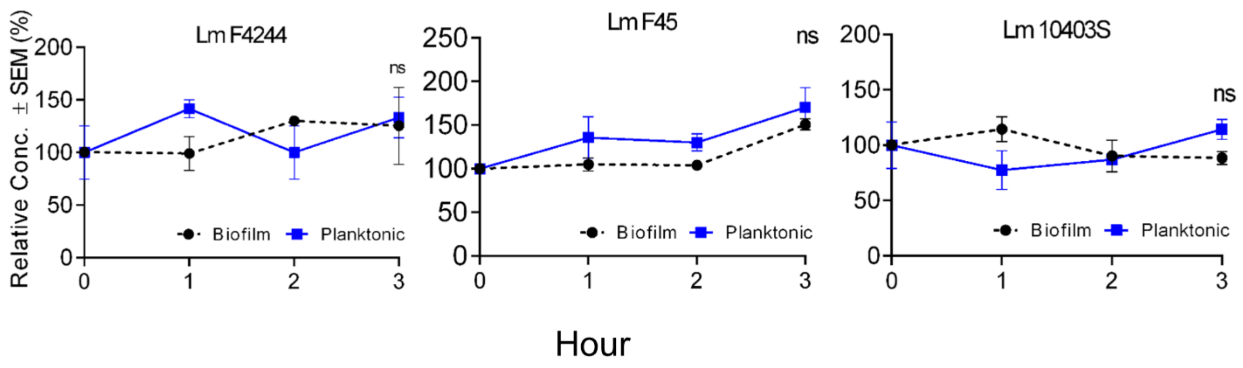

Supplementary Fig. 2. Counts of biofilm-isolated or planktonic *Lm* F4244, F45, and 10403S cells in mammalian cell culture medium (D10F; Dulbecco's modified Eagles medium containing 10% fetal bovine serum) remained very similar over 3 h period.

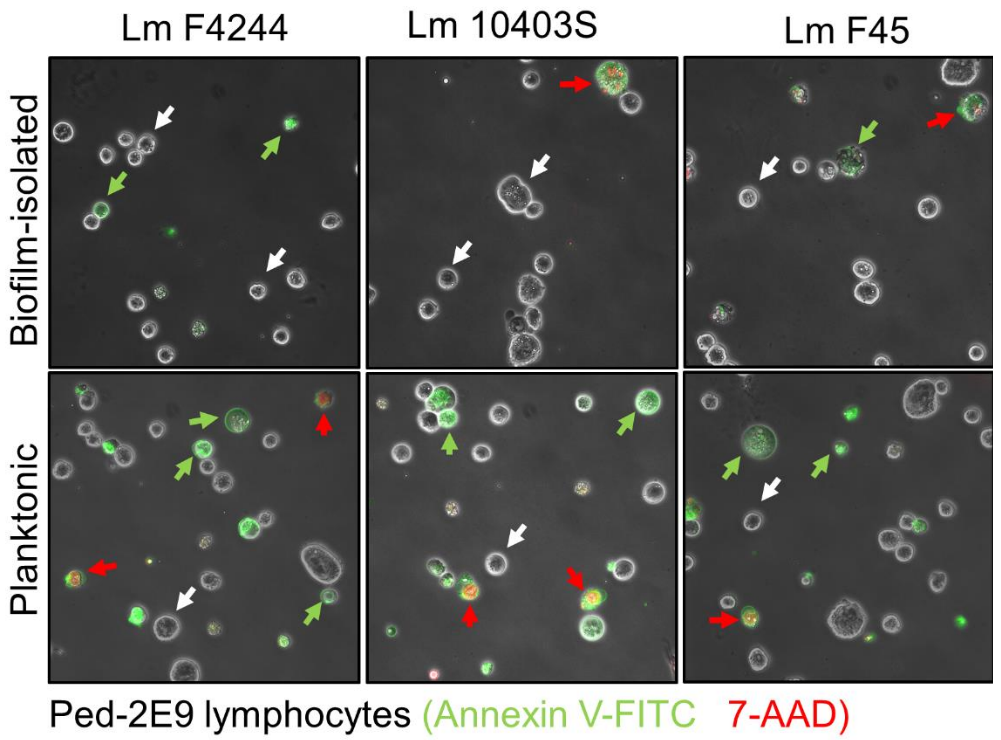

Supplementary Fig. 3. Representative merged fluorescence photomicrograph of Ped-2E9 cells showing pro-apoptotic and apoptotic cells. Live (white arrows) cells, early apoptotic (green arrows), or dead (red arrows) cells were observed after Annexin V-FITC and PI staining.

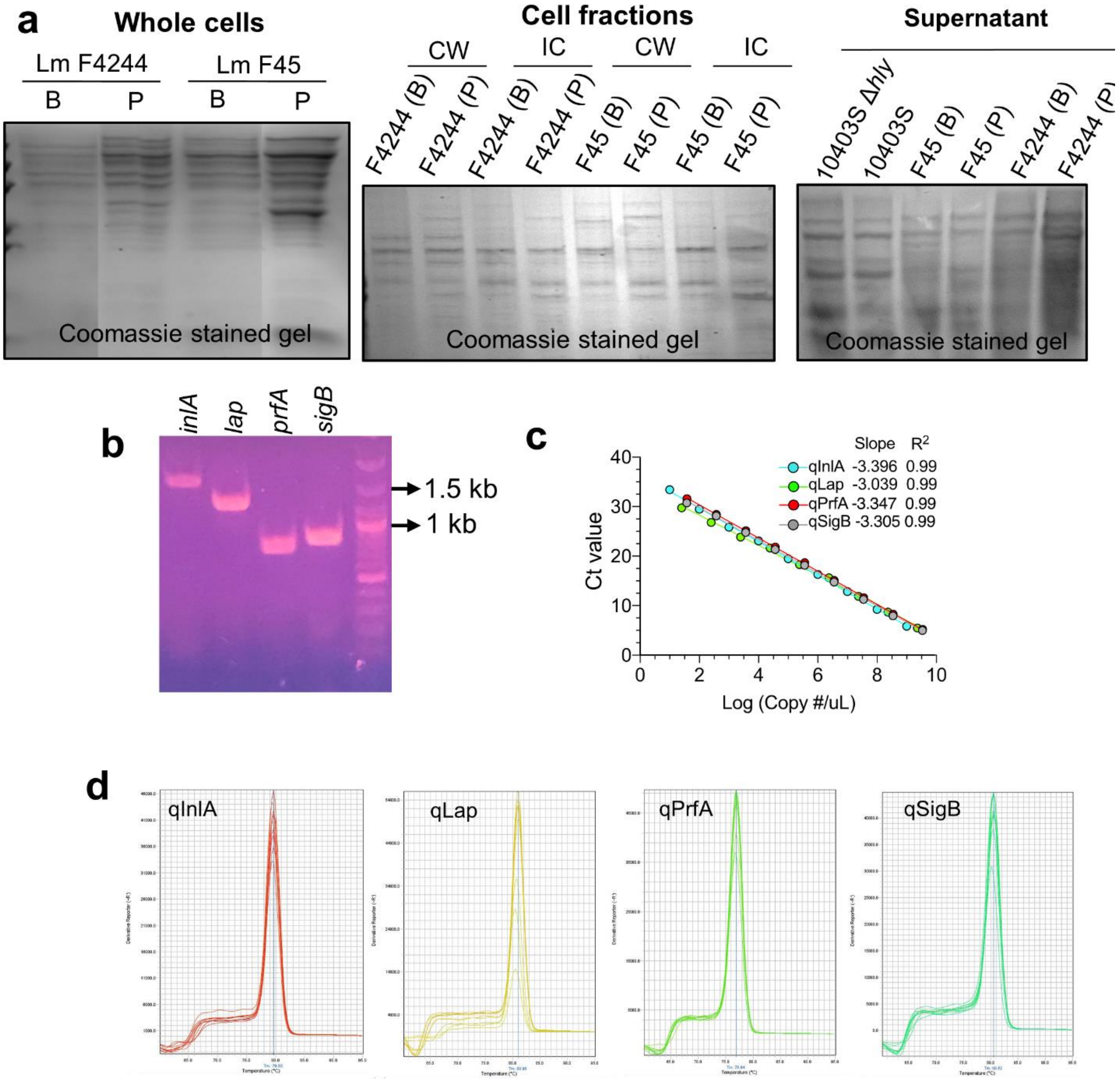

Supplementary Fig 4. (**a**) Coomassie-stained gels loaded with the same protein samples used in **Fig. 4a, 4b,** and **4c,** respectively**.** (**b**) Agarose gel picture showing PCR amplicons of gene *inlA* (1,436 bp), *lap* (1,136 bp), *prfA* (705 bp), and *SigB* (780 bp) that were extracted from the gel (Thermo Fisher Scientific) and used as templates for (**c**) qPCR standard curves. (**d**) Melting curves of respective qPCR amplicons.

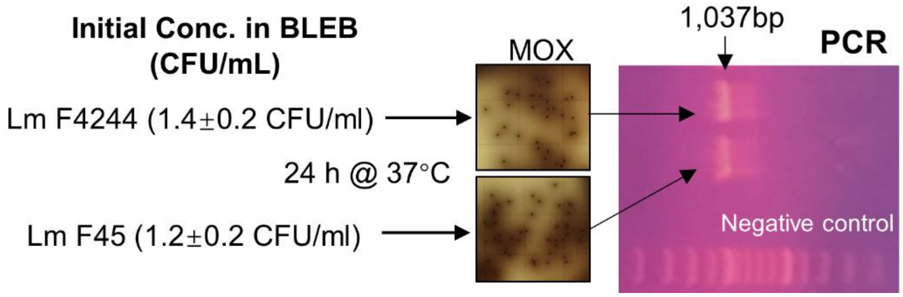

Supplementary Fig 5. Verification of detection of low levels of *Lm*. *Lm* F4244 or F45 were inoculated into BLEB and incubated at 37°C for 24 h. The cultures were inoculated on MOX from where colonies with black centers were verified by PCR using primers targeting InlA (inlAm5 and inlAm3, see Supplementary Table 2) as *Lm.*

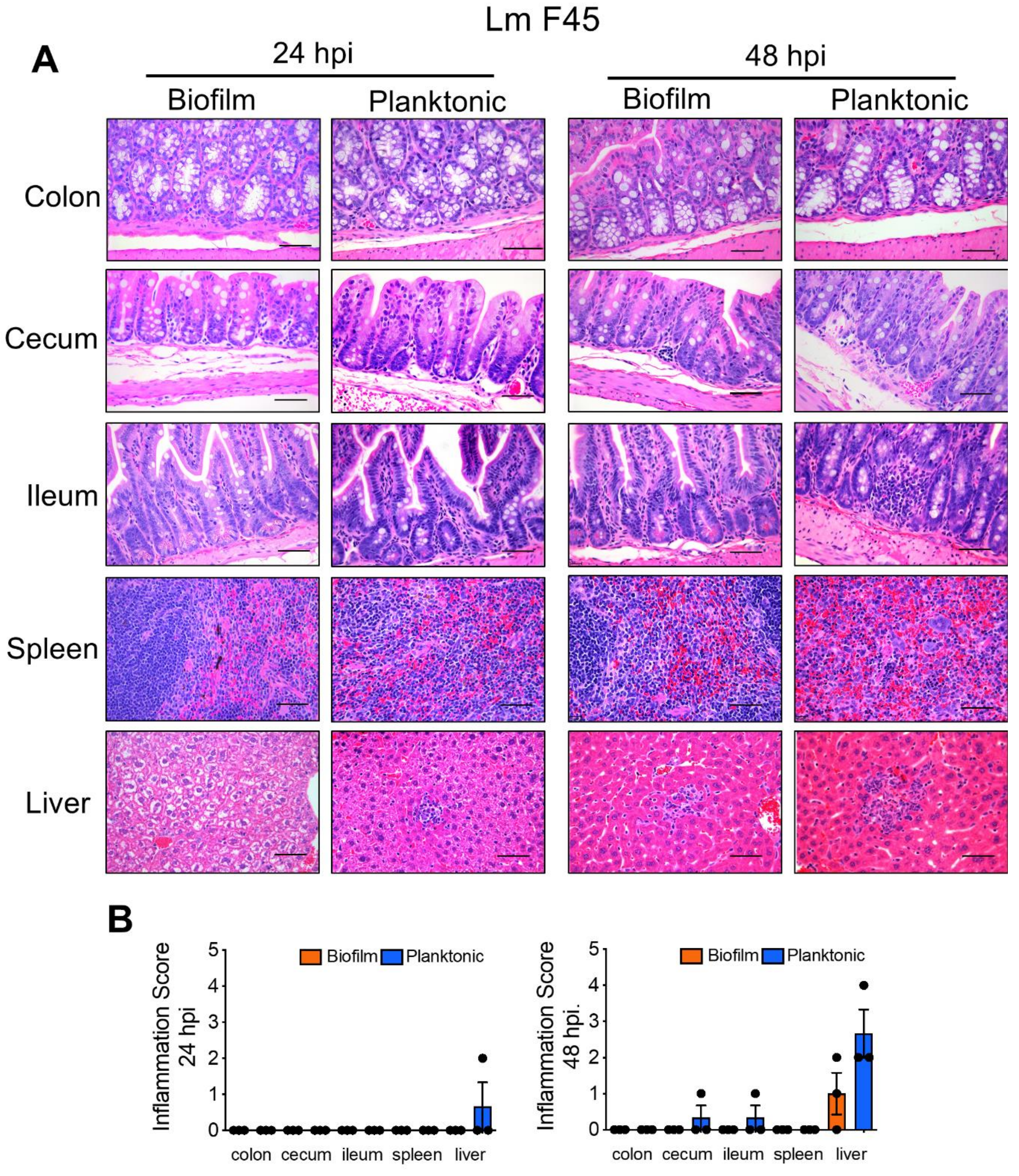

Supplementary Fig. 6. Representative images of hematoxylin and eosin-stained tissue sections of mice challenged with *Lm* F45 sessile (B) or planktonic (P) cells @ 1×10^9 CFU/mouse at 24 and 48 hpi (**A**) and a graph representing histopathological inflammation scores at 24 hpi (**B**, left panel) and 48 hpi (**B,** right panel). Scale bars represent 50 µm.

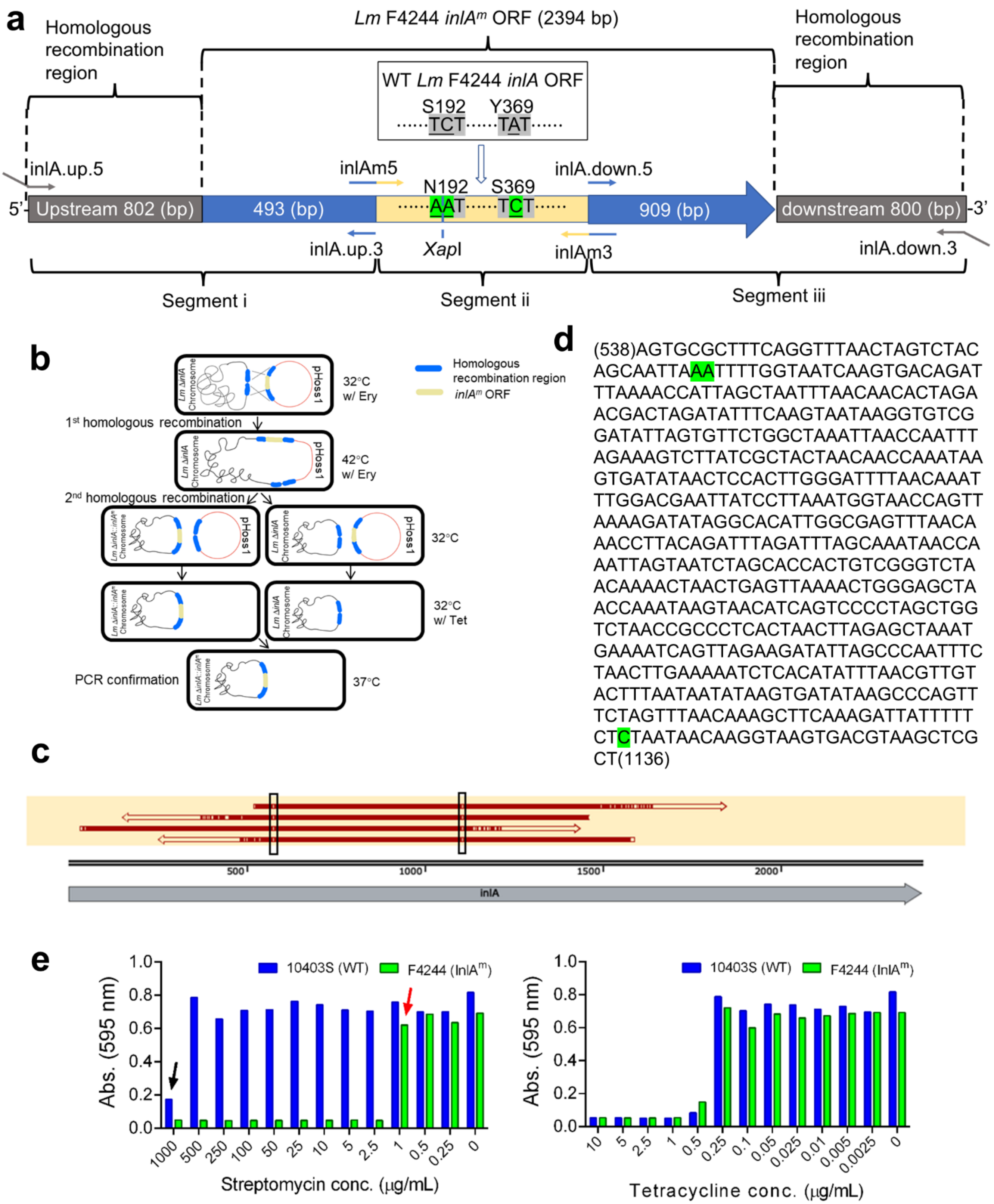

Supplementary Fig. 7. The molecular approach in generating *Lm* F4244 expressing InlA^{m}. (**a**)

Schematic showing the construction of *inlA*^{m} knock-in fragment. Segment ii (yellow), located between nucleotide 494 and 1485 of *Lm* F4244 *inlA* ORF and contains three mutated nucleotides (green), was synthesized by GenScript and amplified using primers inlAm5 and inlAm3 (**Supplementary Table 2**). The mutations resulted in the substitution of amino acids 192 and 369 of InlA from S and Y to N and S, respectively. A *XapI* cutting site was created after the mutation and used for rapid identification. Segment i, the upstream (gray) and beginning regions (blue) of *inlA* ORF, was amplified using WT *Lm* F4244 gDNA as templates and primers inlA.up.5 and inA.up.3 (**Supplementary Table 2**). Segment ii, the ending (blue) and downstream (gray) region, was amplified with primers inlA.down.5 and inlA.down.3 (**Supplementary Table 2**). The three segments were mixed and used as the template to amplify the complete knock-in fragment with *NcoI* and *SalI* sites added to 5' and 3' ends, respectively, using primers inlA.up.5 and inlA.down.3. The knock-in fragment was ligated into pHoss1 and electroporated into *Lm* F4244 Δ*inlA* to insert *inlA*^{m} gene in the chromosome. (**b**) Schematic showing the selection of chromosomal *inlA*^{m} knock-in mutant through two-step homologous recombination. (**c**) Confirmation of mutation in nucleotide sequence (boxed areas, arrows) by Sanger sequencing using four primers of two directions. Red and gray arrows represent the four sequencing results of inlA^{m} and sequence of WT. SnapGene program was used to generate this schematic. (bottom) Nucleotides marked with green represent the mutation site in *inlA*^{m} gene. (**d**) Confirmation of nucleotide sequence showing a mutation in *inlA*^{m} gene. (**e**) Analysis of the sensitivity of *Lm* F4244 InlA^{m} and 10403S (as reference strain) to streptomycin and tetracycline (as control). Two-hundred microliter BHI containing approx. 1×10^{6} CFU/mL of *Lm* 10403S or InlA^{m}BL520 in 96-well microtiter plates were added with serial diluted streptomycin (0.25-1,000 µg/mL) or tetracycline (0.0025-10 µg/mL) and incubated at 37°C for 48 h. Bacterial growth was measured using a microtiter plate reader. *Lm* 10403S was able to grow in 1,000 µg/mL streptomycin (black arrow) while *Lm* F4244 InlA^{m} can grow 1 µg/mL (red arrow), suggesting InlA^{m} is significantly more sensitive to streptomycin than *Lm* 10403S. Meanwhile, the same strains were sensitive to tetracycline when used as a control.

Supplementary Table S1. *Listeria monocytogenes* cultures used in the study
Food-isolates

L. monocytogenes	Serotype	Ribotype	Source[a]
F1	1/2b	DUP-19165	Ground beef, Goias, Brazil, 1990
F2	1/2b	DUP-1042	Chicken, Goias, Brazil,1992
F3	1/2b	DUP-1042	Chicken, Goias, Brazil, 1992
F4	1/2b	DUP-1042	Chicken, Goias, Brazil, 1992
F5	1/2a	DUP-1042	Chicken, Goias, Brazil, 1992
F6	4b	DUP-1042	Chicken, Goias, Brazil, 1992
F7	4b	DUP-18627	Chicken, Goias, Brazil, 1992
F8	4b	DUP-18627	Chicken, Goias, Brazil, 1992
F9	4b	DUP-1042	Chicken, Goias, Brazil, 1993
F10	1/2b	DUP-1042	Chicken, Goias, Brazil, 1993
F11	4b	DUP-1038	Chicken, Goias, Brazil, 1993
F12	4b	DUP-1038	Chicken, Goias, Brazil, 1993
F13	4b	DUP-1042	Chicken, Goias, Brazil, 1993
F14	4b	DUP-1042	Chicken, Goias, Brazil, 1993
F15	4b	DUP-1042	Chicken, Goias, Brazil, 1993
F16	4b	DUP-1042	Chicken, Goias, Brazil, 1993
F17	4b	DUP-1042	Chicken, Goias, Brazil, 1993
F18	4b	DUP-1042	Chicken, Goias, Brazil, 1993
F19	1/2b	DUP-1042	Chicken, Goias, Brazil, 1993
F20	1/2b	DUP-1042	Chicken, Goias, Brazil, 1993
F21	1/2b	DUP-1042	Chicken, Goias, Brazil, 1993
F22	1/2b	DUP-1042	Chicken, Goias, Brazil, 1993
F23	4b	DUP-1038	Chicken, Goias, Brazil, 1993
F24	1/2b	DUP-1042	Chicken, Goias, Brazil, 1993
F25	1/2b	DUP-1042	Chicken, Goias, Brazil, 1993
F26	ND	DUP-1065	Smoked loin, Sao Paulo, Brazil, 2001
F27	1/2b	DUP-18603	Smoked loin, Sao Paulo, Brazil, 2001
F28	4b	DUP-1042	Pizza, Sao Paulo, Brazil, 2001
F29	1/2c	DUP-1051	Sausage, Sao Paulo, Brazil, 2001
F30	4b	DUP-1042	Cooked ham, Minas Gerais, Brazil, 2001
F31	1/2c	DUP-19175	Chicken raw sausage, Minas Gerais, Brazil, 2001
F32	4b	DUP-1042	Pork raw sausage, Minas Gerais, Brazil, 2001
F33	4b	DUP-1042	Mozzarella, Rio Grande do Sul, Brazil, 2002
F34	4b	DUP-18598	Colony cheese, Rio Grande do Sul, Brazil, 2002
F35	4b	DUP-1042	Yellow cheese, Rio Grande do Sul, Brazil, 2002
F36	1/2a	DUP-19174	Frozen cooked beef, Mato Grosso, Brazil, 2005
F37	1/2b	DUP-1046	Cream cheese, Rio de Janeiro, Brazil, 2005
F38	1/2a	DUP-1051	Cheese, Parana, Brazil, 2005

F39	1/2a	DUP-1034	Cheese, Parana, Brazil, 2005
F40	4b	DUP-1042	Ground beef, Sao Paulo, Brazil
F41	1/2a	DUP-19187	Sausage, Sao Paulo, Brazil
F42	1/2c	DUP-1039	Ham, Sao Paulo, Brazil
F43	ND	DUP-15209	Salami, Sao Paulo, Brazil
F44	3c	DUP-1042	Sausage, Sao Paulo, Brazil
F45	1/2b	DUP-1042	Sausage, Sao Paulo, Brazil
F47	1/2c	DUP-19165	Sausage, Sao Paulo, Brazil
F48	3c	DUP-1039	Sausage, Sao Paulo, Brazil
F49	3b	DUP-1042	Ground beef, Sao Paulo, Brazil
F50	4b	DUP-18598	Ground beef, Sao Paulo, Brazil
V7	1/2a	DUP-1039	Raw milk, Massachusetts, USA
V37CE	4b	DUP-1039	Raw milk, Massachusetts, USA
101M	4b	DUP-1044	Salami, USA
103M	1/2a	DUP-1039	Sausage, USA
F4393	4b	DUP-1038	Cheese, USA
ATCC 51414	4b	DUP-1006	Raw milk, Massachusetts, USA
F1057	4b	DUP-1044	Raw milk, USA
F1109	4b	DUP-1044	Raw milk, USA
ATCC 43257	4b	ND	Cheese, California, USA
ATCC 15313	1/2a	ND	Rabbit, Cambridge, England
ATCC 19116	4c	DUP-1061	poultry, USA
ATCC 19118	4e	DUP-1038	poultry USA
ATCC 19114	4a	DUP-1059	bovine brain, USA
ATCC 19117	4d	DUP-1042	sheep, USA
ATCC 19111	½ a	ND	Poultry, England

Clinical-isolates

CHLR1	1	DUP-1023	Blood, newborn female, 1984
SLCC 2482	7	DUP-1042	Patient, feces, Copenhagen, Denmark
ATCC 7644	1/2c	DUP-1039	Patient, CSF, Edenborough, Scotland
ATCC 19112	½ c	DUP-1039	Patient, CSF
Scott A	4b	DUP-1042	Patient, blood, Massachusetts, USA
CHLR10	4	DUP-1038	23-day old female child, meningitis
CHLR8	1	DUP-1053	A 14-day old black female child, meningitis
F4233	½ b	DUP-1042	CDC, patient, CSF/Blood, USA
C12-S(L)	4	ND	Bovine, feces, Nebraska, USA
CHLR6	1	DUP-1042	CHRL, female, meningitis
171	1/2a	DUP-1023	Patient, blood, USA
CHLR9	4	DUP-1038	CSF
CAP	4b	DUP-1038	Patient, CSf, USA
F4260	½ b	DUP-1042	Patient, Blood, USA

ATCC 19115	4b	DUP-1042	Patient, CSF, Germany
F4244	4b	DUP-1044	Patient, CSF, USA
CHLR2	1	DUP-1042	CSF, 2-week old female child
10403S	1/2a	ND	Human skin lesion, USA
ATCC 2540	3b	DUP-1052	Patient, CSF, New Orleans, USA
CHLR7	4	DUP-1024	Human
H6	4b	DUP-18604	CSF, 26-year-old patient, Sao Paulo, 2001
CHLR5	4	DUP-1042	CSF, 11-day old male child
CHLR3	4	DUP-1038	CSF, 2-week old female child
H11	1/2b	DUP-19175	Blood, 72 years old patient, Sao Paulo, 2005
H9	4b	DUP-1038	Blood, 48 years old patient, Sao Paulo, 2004
H1	4b	DUP-1038	Aorta prosthesis, 69 years old patient, Sao Paulo, 2001
Murray B	4ab	DUP-1042	Patient, Massachusetts, USA
H5	1/2a	DUP-1023	CSF, 71 years old patient, Sao Paulo, 1995
H8	4b	DUP-1042	Blood, 61 years old patient, Sao Paulo, 2003
H16	ND	ND	Blood, 6 days old patient, Sao Paulo, 1998
F4243	4b	ND	Patient, CSF, USA
H12	4b	DUP-1038	CSF, Sao Paulo, 2005
H14	ND	DUP-1042	CSF, Sao Paulo, 2002
H10	1/2a	DUP-19173	CSF, 55 years old patient, Sao Paulo, 2004
H13	ND	DUP-1042	CSF, 16 years old patient, Sao Paulo, 2002
F4263	½ a	DUP-1060	Patient, CSF/Blood, USA
F4264	4b	DUP-1038	Patient, CSF/Blood, USA
H7	4b	DUP-1042	Blood, 5 days old patient, Sao Paulo, 2004
SLCC 2482	7	DUP-1042	VICAM, patient, feces, Copenhagen, Denmark
H15	1/2b	DUP-1042	CSF, Sao Paulo, 2005
F4262	4b	DIP-1051	CDC, patient, CSF/Blood, USA
H4	4b	DUP-1042	Blood, 34 years old patient, Sao Paulo, 1998
H3	4b	DUP-19191	CSF, 60 years old patient, Sao Paulo, 1998
ATCC 19113	3a	DUP-1030	Patient, Copenhagen, Denmark
H2	4b	DUP-18604	CSF, 73 years old patient, Sao Paulo, 2000
ATCC 19115	4b	DUP-1042	VICAM, patient, CSF, Germany

[a]CSF, Cerebrospinal fluid; ATCC, American Type Culture Collection; CHLR, Children's Hospital at Little Rock, Arkansas, USA. ND, not determined.

Supplementary Table 2. Plasmid, primers, and antibodies used in the study

qPCR Oligonucleotides Primers	Sequence	Source
pHoss1	Available at Addgene.org	Addgene
inlAm5	AGACCCGCTTAAAAACCTAACAAATTTAAATCGGCTAGAACTATCT	This study
inlAm3	GAAACTTAGCATTAAAAATTGCCTTAAGTGGCTGCGTCACTGT	This study
inlA.up.5 (*NcoI*)	cacaagggtttgaatcattagatcccatggATCCGATTATTGTAGTGGCTT	This study
inlA.up.3	ATAGTTCTAGCCGATTTAAATTTGTTAGGTTTTTAAGCGGGTCT	This study
inlA.down.5	CGCAGCCACTTAAGGCAATTTTTAATGCTAAGTTTCA	This study
inlA.down.3 (*SalI*)	gatatcggatccatatgacgtcgacCTAAACAATTCTAAAACA	This study
InlA_3(RT)[a]	GCATTATAGCTATCGCCAGT	This study
inlA_5(RT)[a]	ACAAAACTAACTGAGTTAAAACTGG	
qInlA_3[b]	GTTGTTACACCGTCATTATCCAAGGTTGCTG	This study
qInlA_5[b]	ATTGACTGAACCAGCTAAGCCTGTAAAAGAAGG	
Lap_3(RT)[a]	TCAAACACCTTTGTAAGCTT	This study
Lap_5(RT)[a]	GAACGCGTATTTATCGTAACT	
qLap_3[b]	CGCATTTGCAAACGCCATACCAGC	This study
qLap_5[b]	CCAGATGTTGCGATTGTCGATGCAC	
prfA_3(RT)[a]	TTTTCCCCAAGTAGCAGG	This study
prfA_5(RT)[a]	ATGAACGCTCAAGCAGAA	
qprfA_3[b]	GCTAGGCTGTATGAAACTTGTTTTTGTAGG	This study
qprfA_5[b]	AGAAGTCATTAGCGAACAGGCTACCGC	
SigB_3(RT)[a]	TTACTCCACTTCCTCATTCTG	This study
SigB_5(RT)[a]	ATGCCAAAAGTATCTCAACC	
qSigB_3[b]	CCCATTTCCATTGCTTCTAAAACTTCTTCCTCC	This study
qSigB_5[b]	AATTAGGTCCGAAAATTAAAAATGCCGTAGAAGAG	
inlA-F[c]	GAACCAGCTAAGCCCGTAAAAG	(Werbrouck et al. 2006)
inlA-R[c]	CGCCTGTTTGGGCATCA	
prfA-F[c]	TCATTAGCGAGCAGGCTACC	(Camejo et al. 2009)
prfA-R[c]	GCAAATAGAGCCAAGCTTCC	

**Antibodies**		**Source**
Mouse monoclonal anti-LAP (mAb-H7)		Our lab
Mouse monoclonal anti-InlA (mAb-2D12)		(Mendonca et al. 2012)
Rabbit polyclonal anti-LLO		Abcam (Cat # ab200538)
Goat anti-rabbit IgG (HRP-linked)		Cell Signaling (Cat # 7074)
Horse anti-mouse IgG (HRP-linked)		Cell Signaling (Cat # 7076)

[a]Primers used to generate amplicons for generating qPCR standard curves. [b]qPCR primers used for quantifying gene copy numbers. [c]qPCR primers are used for detecting the presence or absence of *Lm* in mouse tissues.

Supplementary Table 3. Confirmation of mouse tissue/organ (12 hpi) for *L. monocytogenes* by qPCR

	**Ct values, Biofilm-isolated *Lm* F4244**										**Ct values, Planktonic *Lm* F4244**											
Mouse #	1		2		3		4		5		1		2		3		4		5		6	
Target gene	*inlA*	*prfA*	*inlA*	*prfA*	*inlA*	*prfA*	*inlA*	*prfA*	*inlA*	*prfA*	*inlA*	*prfA*	*inlA*	*prfA*	*inlA*	*prfA*	*inlA*	*prfA*	*inlA*	*prfA*	*inlA*	*prfA*
Jejunum	31 (-)	32 (-)	31 (-)	31 (-)	31 (-)	33 (-)	31 (-)	29 (-)	31 (-)	31 (-)	31 (-)	32 (-)	32 (-)	31 (-)	30 (-)	31 (-)	32 (-)	31 (-)	NT	NT	NT	NT
Ileum	33 (-)	32 (-)	31 (-)	32 (-)	30 (-)	32 (-)	31 (-)	32 (-)	32 (-)	31 (-)	33 (-)	32 (-)	31 (-)	31 (-)	32 (-)	30 (-)	29 (-)	32 (-)	NT	NT	NT	NT
Cecum	31 (-)	33 (-)	13 (+)	13 (+)	31 (-)	31 (-)	30 (-)	32 (-)	32 (-)	30 (-)	13 (+)	13 (+)	12 (+)	13 (+)	13 (+)	12 (+)	13 (+)	12 (+)	NT	NT	NT	NT
Colon	33 (-)	32 (-)	12 (+)	13 (+)	32 (-)	31 (-)	29 (-)	30 (-)	29 (-)	30 (-)	11 (+)	13 (+)	33 (-)	30 (-)	13 (+)	12 (+)	31 (-)	31 (-)	NT	NT	NT	NT
MLN	31 (-)	31 (-)	32 (-)	32 (-)	32 (-)	31 (-)	30 (-)	32 (-)	31 (-)	30 (-)	29 (-)	32 (-)	11 (+)	12 (+)	13 (+)	12 (+)	11 (+)	12 (+)	31 (-)	31 (-)	31 (-)	32 (-)
Spleen	31 (-)	32 (-)	31 (-)	31 (-)	31 (-)	32 (-)	32 (-)	29 (-)	32 (-)	32 (-)	31 (-)	32 (-)	31 (-)	29 (-)	12 (+)	13 (+)	32 (-)	30 (-)	31 (-)	32 (-)	31 (-)	31 (-)
Liver	32 (-)	32 (-)	30 (-)	31 (-)	30 (-)	31 (-)	30 (-)	29 (-)	31 (-)	30 (-)	12 (+)	13 (+)	31 (-)	29 (-)	31 (-)	31 (-)	30 (-)	32 (-)	11 (+)	12 (+)	13 (+)	13 (+)
Kidney	32 (-)	31 (-)	31 (-)	31 (-)	30 (-)	31 (-)	32 (-)	32 (-)	31 (-)	32 (-)	30 (-)	31 (-)	31 (-)	30 (-)	30 (-)	31 (-)	31 (-)	32 (-)	31 (-)	32 (-)	30 (-)	32 (-)

	**Ct values, Biofilm-isolated *Lm* F45**										**Ct values, planktonic *Lm* F45**									
Mouse #	1		2		3		4		5		1		2		3		4		5	
Target gene	*inlA*	*prfA*	*inlA*	*prfA*	*inlA*	*prfA*	*inlA*	*prfA*	*inlA*	*prfA*	*inlA*	*prfA*	*inlA*	*prfA*	*inlA*	*prfA*	*inlA*	*prfA*	*inlA*	*prfA*
Jejunum	29 (-)	28 (-)	30 (-)	31 (-)	30 (-)	30 (-)	29 (-)	30 (-)	29 (-)	30 (-)	30 (-)	31 (-)	32 (-)	30 (-)	29 (-)	30 (-)	31 (-)	30 (-)	31 (-)	30 (-)
Ileum	30 (-)	32 (-)	29 (-)	30 (-)	28 (-)	30 (-)	31 (-)	29 (-)	31 (-)	30 (-)	29 (-)	31 (-)	30 (-)	32 (-)	31 (-)	29 (-)	29 (-)	31 (-)	30 (-)	29 (-)
Cecum	30 (-)	29 (-)	31 (-)	32 (-)	30 (-)	31 (-)	28(-)	28(-)	30 (-)	29 (-)	17 (+)	19 (+)	31 (-)	30 (-)	19 (+)	14 (+)	19 (+)	19 (+)	15 (+)	16 (+)
Colon	29 (-)	29 (-)	32 (-)	29 (-)	31 (-)	31 (-)	32 (-)	29 (-)	31 (-)	31 (-)	15 (+)	15 (+)	18 (+)	18 (+)	16 (+)	16 (+)	15 (+)	14 (+)	11(+)	11(+)
MLN	32 (-)	31 (-)	32 (-)	30 (-)	30 (-)	31 (-)	31 (-)	32 (-)	30 (-)	30 (-)	29 (-)	30 (-)	16 (+)	17 (+)	29 (-)	30 (-)	28 (-)	30 (-)	14 (+)	15 (+)
Spleen	31 (-)	31 (-)	31 (-)	28 (-)	29 (-)	32 (-)	30 (-)	28 (-)	30 (-)	32 (-)	17 (+)	19 (+)	31 (-)	30 (-)	16 (+)	16 (+)	37 (-)	33 (-)	12 (+)	11 (+)
Liver	30 (-)	31 (-)	31 (-)	31 (-)	30 (-)	31 (-)	29 (-)	29 (-)	29 (-)	30 (-)	13 (+)	15 (+)	30 (-)	29 (-)	29 (-)	30 (-)	30 (-)	31 (-)	13 (+)	14 (+)
Kidney	31 (-)	31 (-)	30 (-)	30 (-)	31 (-)	30 (-)	31 (-)	31 (-)	31 (-)	30 (-)	33 (-)	31 (-)	32 (-)	33 (-)	29 (-)	31 (-)	31 (-)	31 (-)	30 (-)	31 (-)

	**Ct values, Biofilm-isolated *Lm* F4244 InlA^{m}**										**Ct values, planktonic *Lm* F4244 InlA^{m}**									
Mouse #	1		2		3		4		5		1		2		3		4		5	
Target gene	*inlA*	*prfA*	*inlA*	*prfA*	*inlA*	*prfA*	*inlA*	*prfA*	*inlA*	*prfA*	*inlA*	*prfA*	*inlA*	*prfA*	*inlA*	*prfA*	*inlA*	*prfA*	*inlA*	*prfA*
Jejunum	30 (-)	28 (-)	30 (-)	28 (-)	30 (-)	30 (-)	12 (+)	15 (+)	14 (+)	15 (+)	30 (-)	31 (-)	15 (+)	15 (+)	29 (-)	30 (-)	14 (+)	15 (+)	31 (-)	30 (-)

Ileum	28 (-)	28 (-)	29 (-)	29 (-)	29 (-)	30 (-)	31 (-)	30 (-)	30 (-)	30 (-)	31 (-)	30 (-)	15 (+)	14 (+)	31 (-)	29 (-)	29 (-)	31 (-)	15 (+)	16 (+)
Cecum	31 (-)	30 (-)	30 (-)	31 (-)	31 (-)	31 (-)	29 (-)	28 (-)	30 (-)	30 (-)	17 (+)	16 (+)	31 (-)	30 (-)	17 (+)	14 (+)	18 (+)	19 (+)	15 (+)	15 (+)
Colon	30 (-)	29 (-)	31 (-)	29 (-)	29 (-)	30 (-)	32 (-)	30 (-)	31 (-)	31 (-)	14 (+)	15 (+)	17 (+)	17 (+)	16 (+)	17 (+)	31 (-)	29 (-)	31 (-)	29 (-)
MLN	31 (-)	28 (-)	31 (-)	31 (-)	28 (-)	30 (-)	32 (-)	32 (-)	31 (-)	30 (-)	16 (+)	15 (+)	30 (-)	31 (-)	29 (-)	29 (-)	30 (-)	30 (-)	15 (+)	15 (+)
Spleen	32 (-)	32 (-)	30 (-)	32 (-)	32 (-)	32 (-)	30 (-)	30 (-)	30 (-)	31 (-)	14 (+)	15 (+)	30 (-)	30 (-)	15 (+)	15 (+)	32 (-)	32 (-)	13 (+)	13 (+)
Liver	30 (-)	28 (-)	30 (-)	31 (-)	31 (-)	31 (-)	29 (-)	29 (-)	30 (-)	30 (-)	31 (-)	29 (-)	13 (+)	13 (+)	14 (+)	15 (+)	16 (+)	16 (+)	29 (-)	32 (-)
Kidney	31 (-)	31 (-)	31 (-)	31 (-)	30 (-)	30 (-)	29 (-)	29 (-)	31 (-)	31 (-)	31 (-)	31 (-)	32 (-)	32 (-)	31 (-)	29 (-)	31 (-)	31 (-)	30 (-)	30 (-)
Mouse #	6		7		8		9				6		7		8		9			
Target gene	*inlA*	*prfA*	*inlA*	*prfA*	*inlA*	*prfA*	*inlA*	*prfA*			*inlA*	*prfA*	*inlA*	*prfA*	*inlA*	*prfA*	*inlA*	*prfA*		
Jejunum	29 (-)	28 (-)	15 (+)	15 (+)	30 (-)	30 (-)	29 (-)	30 (-)			16 (+)	16 (+)	16 (+)	15 (+)	30 (-)	30 (-)	14 (+)	14 (+)		
Ileum	31 (-)	31 (-)	33 (-)	30 (-)	15 (+)	14 (+)	30 (-)	29 (-)			31 (-)	32 (-)	29 (-)	29 (-)	29 (-)	31 (-)	30 (-)	29 (-)		
Cecum	30 (-)	31 (-)	31 (-)	32 (-)	15 (+)	16 (+)	30(-)	28(-)			13 (+)	14 (+)	14 (+)	14 (+)	29 (-)	31 (-)	29(-)	28(-)		
Colon	28 (-)	29 (-)	30 (-)	30 (-)	13 (+)	15 (+)	14 (+)	14 (+)			30 (-)	29 (-)	30 (-)	29 (-)	14 (+)	14 (+)	14 (+)	15 (+)		
MLN	32 (-)	32 (-)	32 (-)	30 (-)	31 (-)	31 (-)	32 (-)	31 (-)			30 (-)	31 (-)	31 (-)	30 (-)	30 (-)	30 (-)	31 (-)	30 (-)		
Spleen	31 (-)	31 (-)	31 (-)	31 (-)	30 (-)	32 (-)	30 (-)	30 (-)			14 (+)	15 (+)	15 (+)	15 (+)	30 (-)	30 (-)	31 (-)	31 (-)		
Liver	31 (-)	31 (-)	30 (-)	31 (-)	13 (+)	14 (+)	14 (+)	15 (+)			31 (-)	31 (-)	30 (-)	31 (-)	14 (+)	14 (+)	15 (+)	14 (+)		
Kidney	31 (-)	31 (-)	31 (-)	31 (-)	30 (-)	30 (-)	31 (-)	30 (-)			31 (-)	31 (-)	30 (-)	30 (-)	31 (-)	31 (-)	31 (-)	31 (-)		

*Each Ct value is the average of two replicate qPCR. +, positive; -, negative

Supplementary Table 4. *L. monocytogenes* mutant strains and growth conditions

L. monocytogenes	**Incubation conditions**	**Source**
F4244 (WT, 4b)	37°C	Our Lab
BL520 (F4244 *inlA*^{m})	37°C	This study
KB208 (F4244 *lap*$^{-}$)	Erythromycin (10 μg/mL), 42°C	Our Lab (Jagadeesan et al. 2010)
AKB301 (F4244 Δ*inlA*)	37°C	Our Lab (Burkholder and Bhunia 2010)
10403S (WT, 1/2a)	37°C	Dr. Daniel Portnoy, UC Berkley, USA
10403S Δ*prfA*	37°C	Dr. Nancy Freitag, University of Illinois, Chicago, USA
10403S Δ*hly*	37°C	Dr. Daniel Portnoy, UC Berkley, USA

Supplementary References

Burkholder, K.M. and Bhunia, A.K. (2010) *Listeria monocytogenes* uses *Listeria* adhesion protein (LAP) to promote bacterial transepithelial translocation, and induces expression of LAP receptor Hsp60. *Infect Immun* **78**, 5062-5073.

Camejo, A., Buchrieser, C., Couvé, E., Carvalho, F., Reis, O., Ferreira, P., Sousa, S., Cossart, P. and Cabanes, D. (2009) In vivo transcriptional profiling of *Listeria monocytogenes* and mutagenesis identify new virulence factors involved in infection. *Plos Pathog* **5**, e1000449.

Jagadeesan, B., Koo, O.K., Kim, K.P., Burkholder, K.M., Mishra, K.K., Aroonnual, A. and Bhunia, A.K. (2010) LAP, an alcohol acetaldehyde dehydrogenase enzyme in *Listeria* promotes bacterial adhesion to enterocyte-like Caco-2 cells only in pathogenic species. *Microbiology* **156**, 2782-2795.

Mendonca, M., Conrad, N., Conceicao, F., Moreira, A., da Silva, W., Aleixo, J. and Bhunia, A. (2012) Highly specific fiber optic immunosensor coupled with immunomagnetic separation for detection of low levels of *Listeria monocytogenes* and *L. ivanovii*. *BMC Microbiol* **12**, 275.

Werbrouck, H., Grijspeerdt, K., Botteldoorn, N., Van Pamel, E., Rijpens, N., Van Damme, J., Uyttendaele, M., Herman, L. and Van Coillie, E. (2006) Differential inlA and inlB expression and interaction with human intestinal and liver cells by *Listeria monocytogenes* strains of different origins. *Appl Environ Microbiol* **72**, 3862.

Original blots presented in the manuscript

Figure 4a

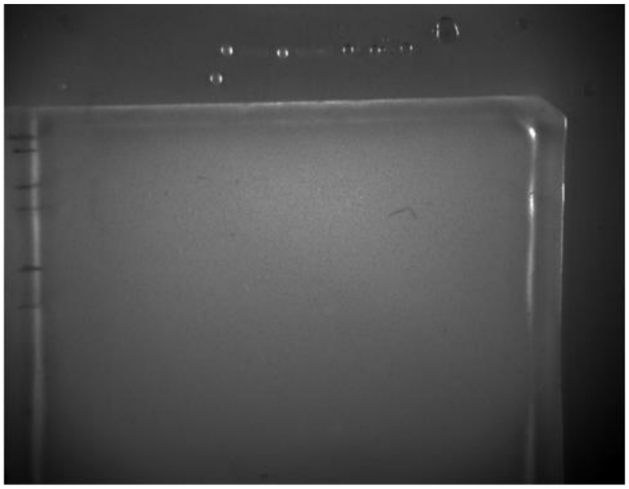

Membrane

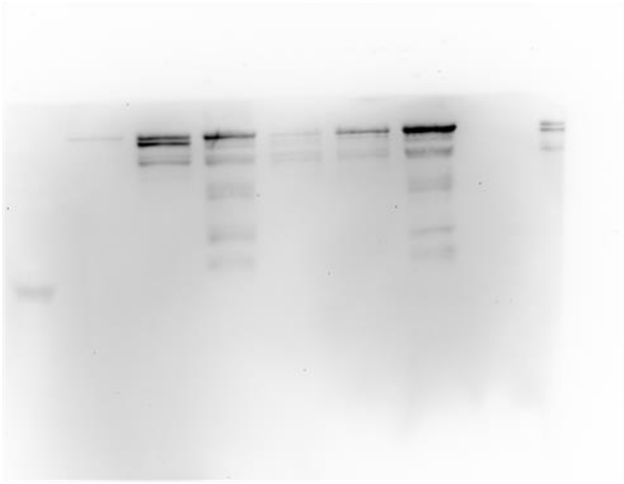

Anti-InlA mAb blot

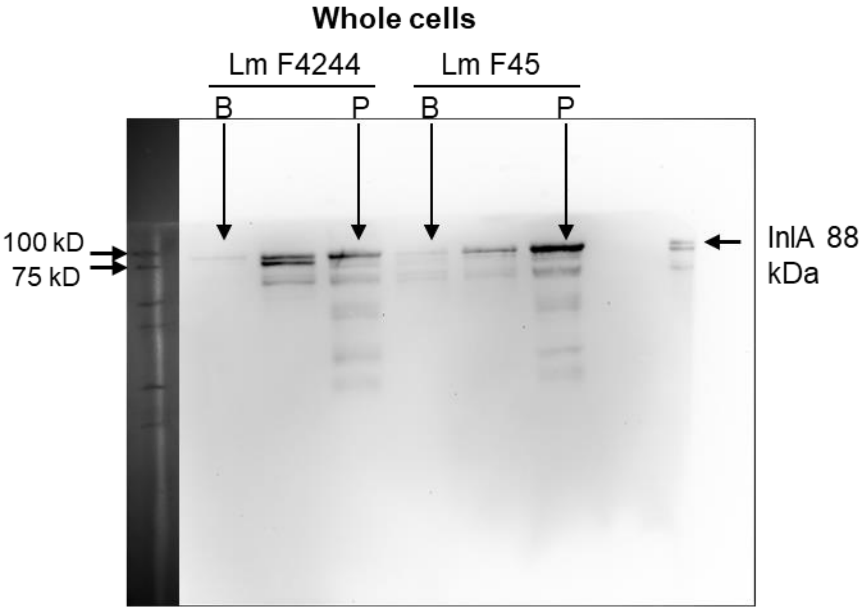

Merged

Figure 4a

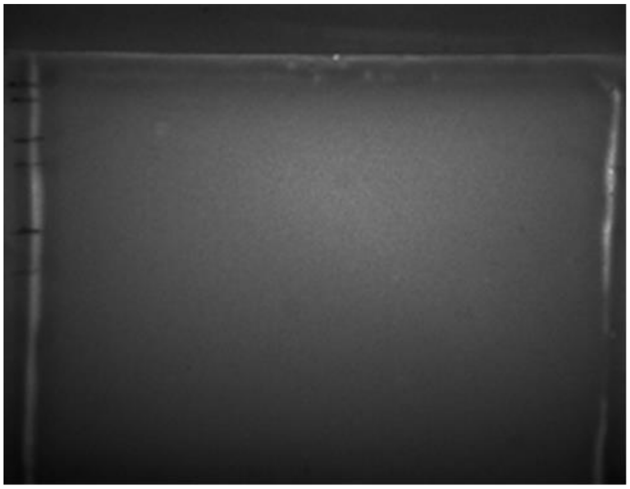

Membrane

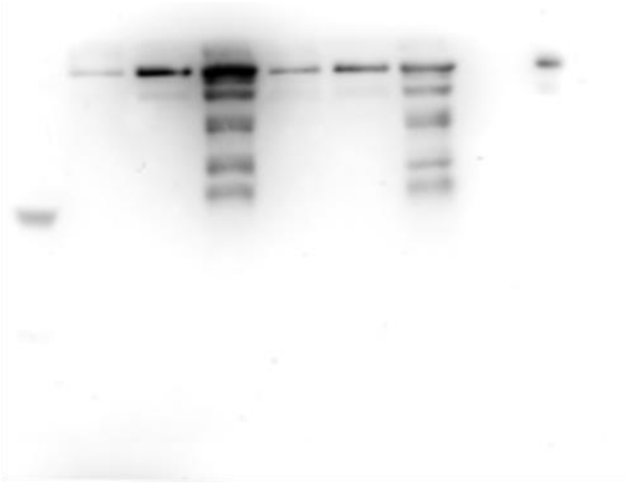

Anti-LAP mAb blot

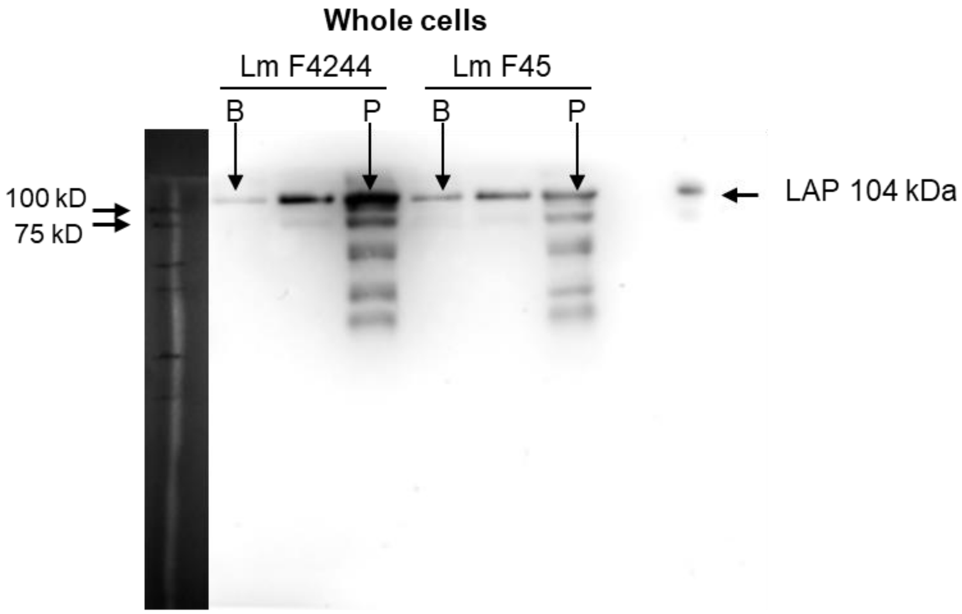

Merged

Figure 4b

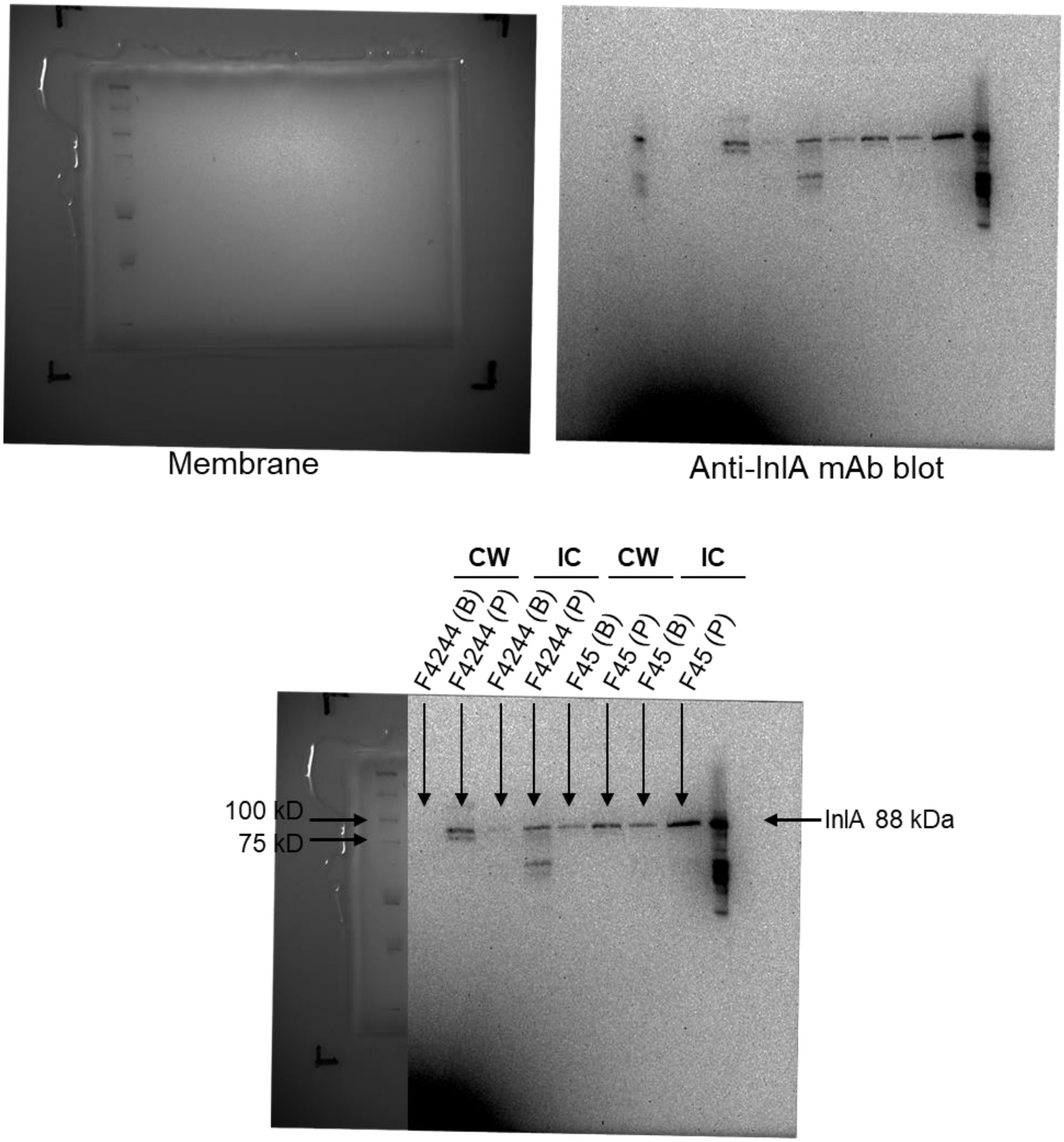

Merged

Figure 4b

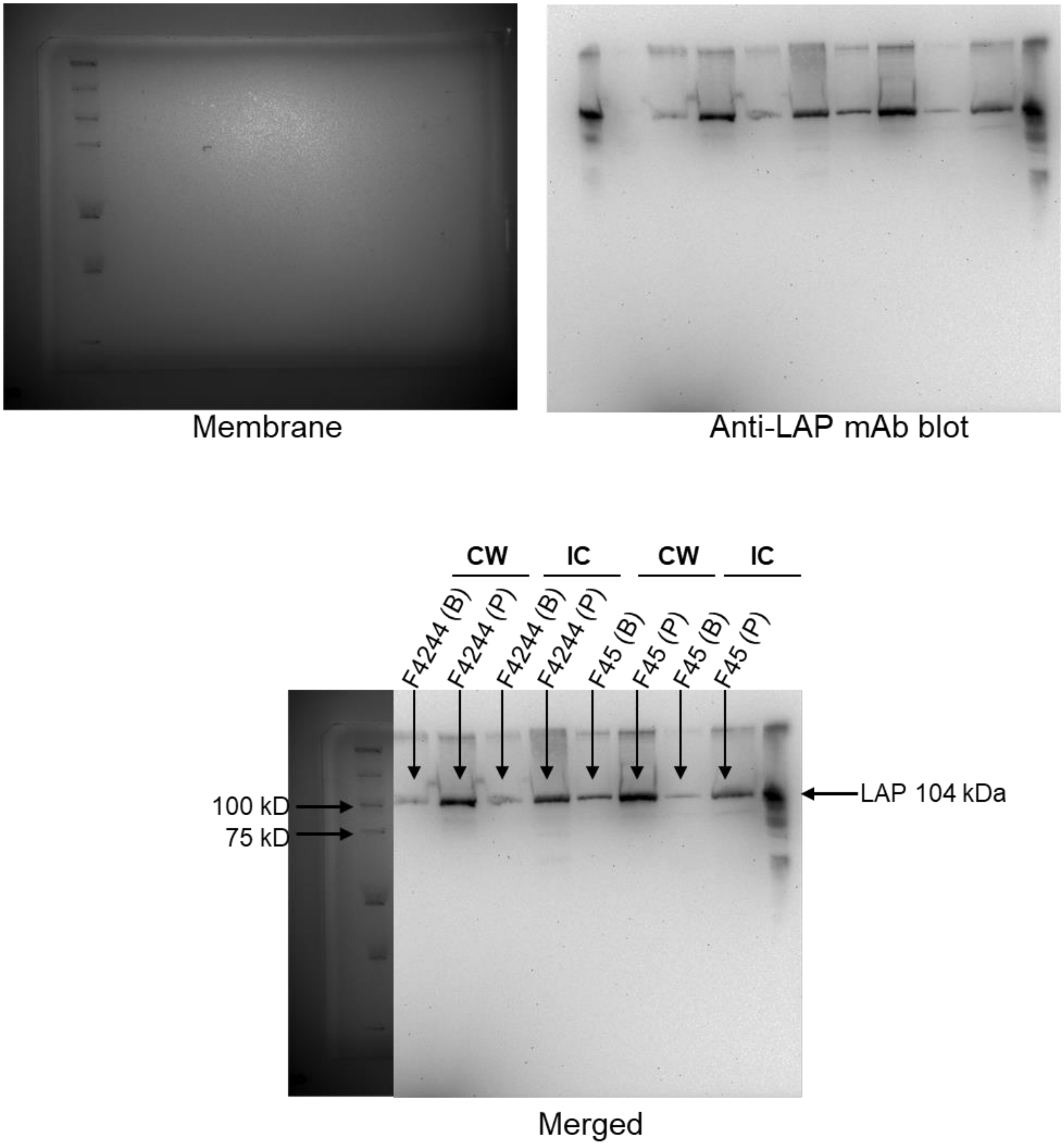

Membrane
Anti-LAP mAb blot
CW
IC
CW
IC
F4244 (B)
F4244 (P)
F4244 (B)
F4244 (P)
F45 (B)
F45 (P)
F45 (B)
F45 (P)
100 kD
75 kD
LAP 104 kDa
Merged

Figure 4c

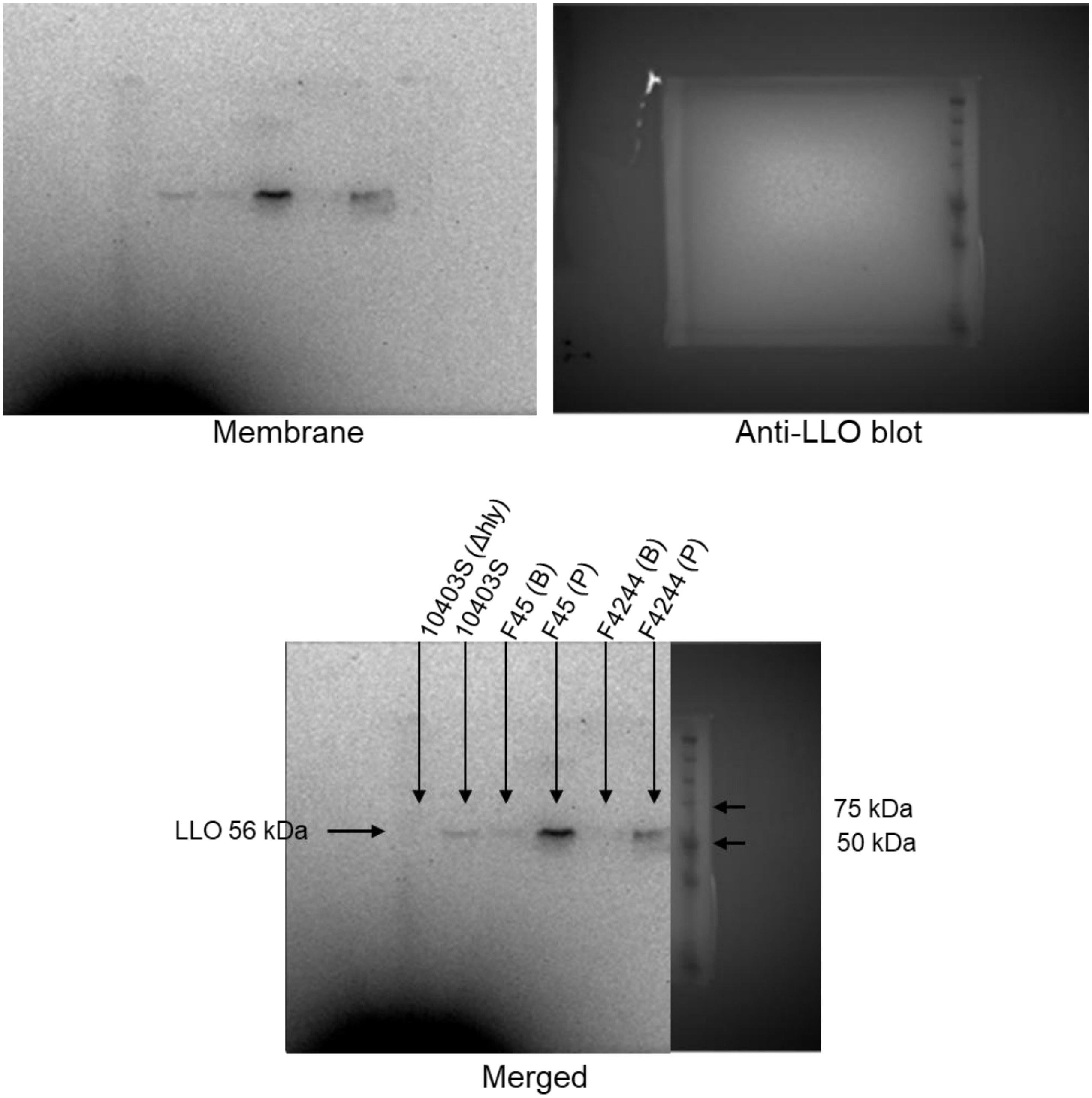
Membrane
Anti-LLO blot
10403S (Δhly)
10403S
F45 (B)
F45 (P)
F4244 (B)
F4244 (P)
LLO 56 kDa
75 kDa
50 kDa
Merged

Figure 7b

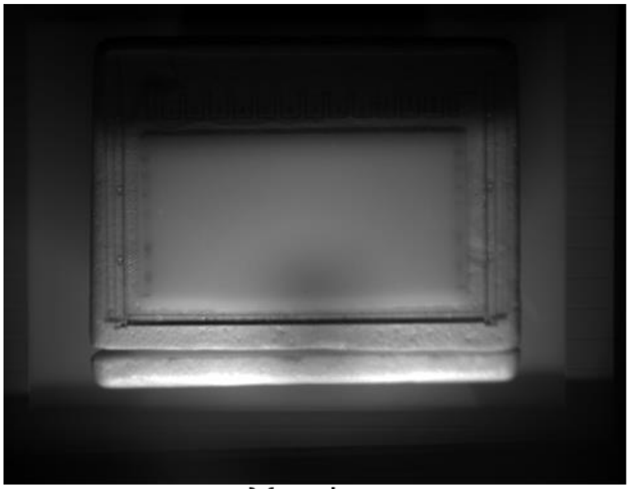

Membrane

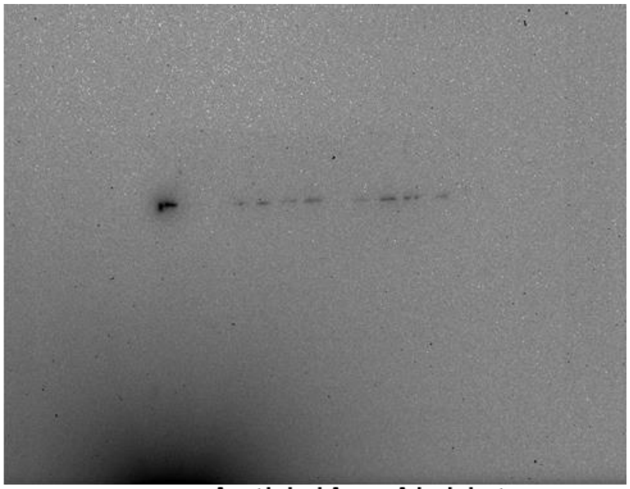

Anti-InlA mAb blot

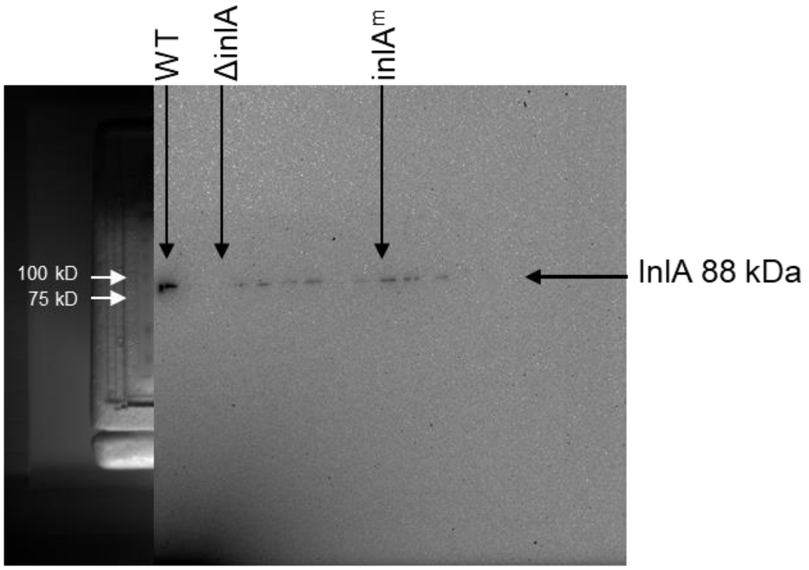

Merged

Figure 7b

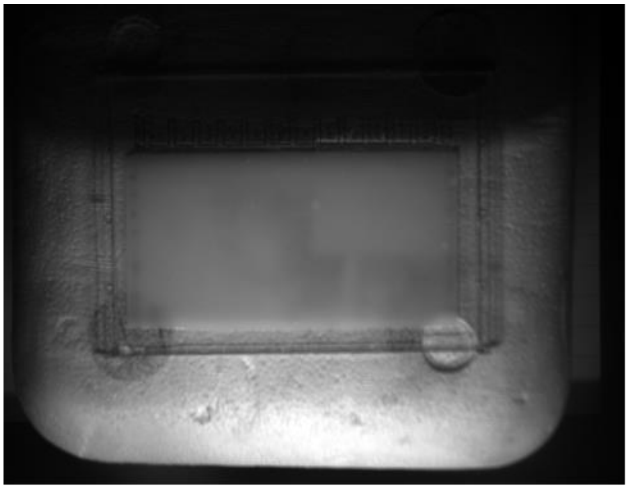

Membrane

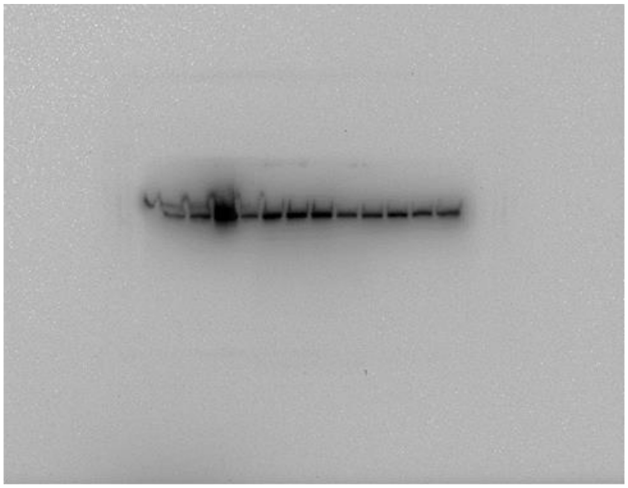

Anti-LAP mAb blot

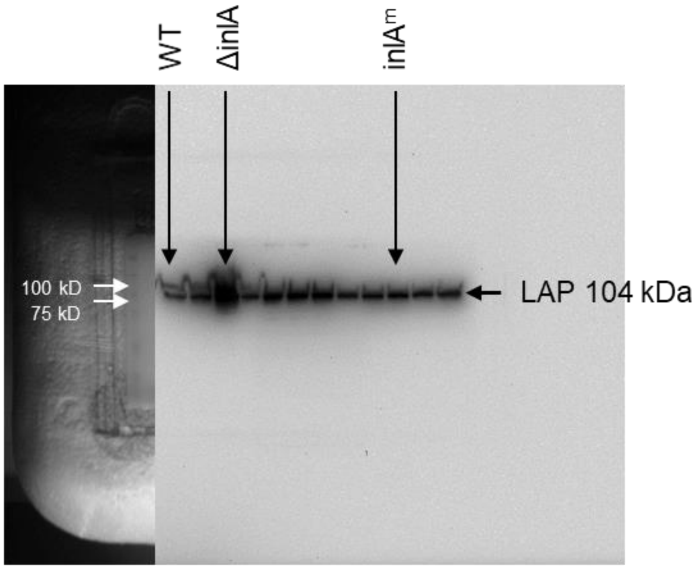

Merged

Figure 8e

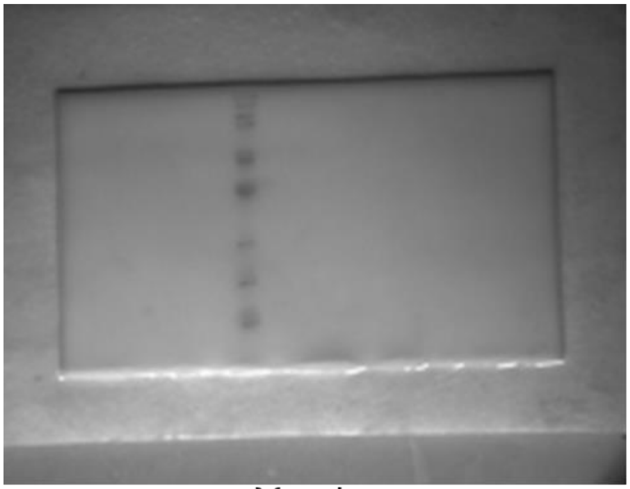

Membrane

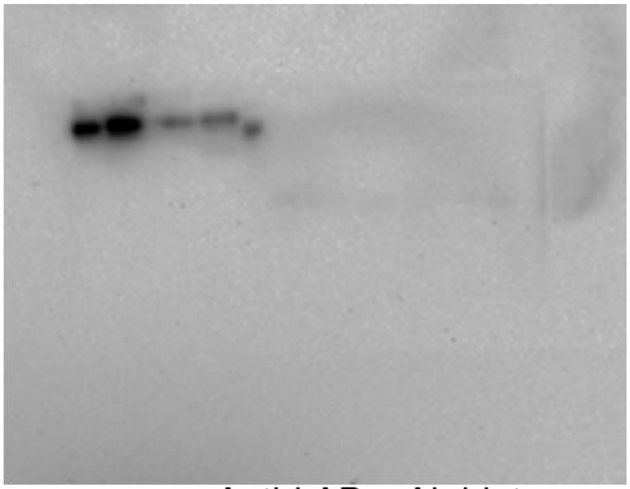

Anti-LAP mAb blot

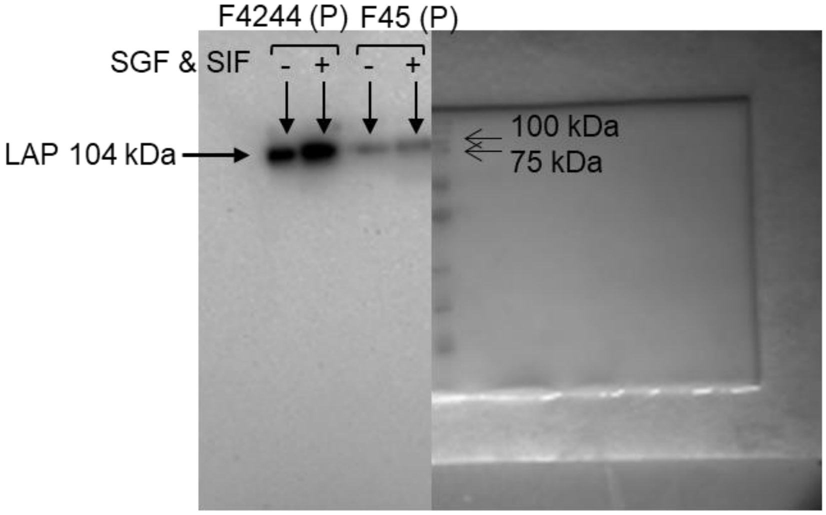

Merged

Figure 8e

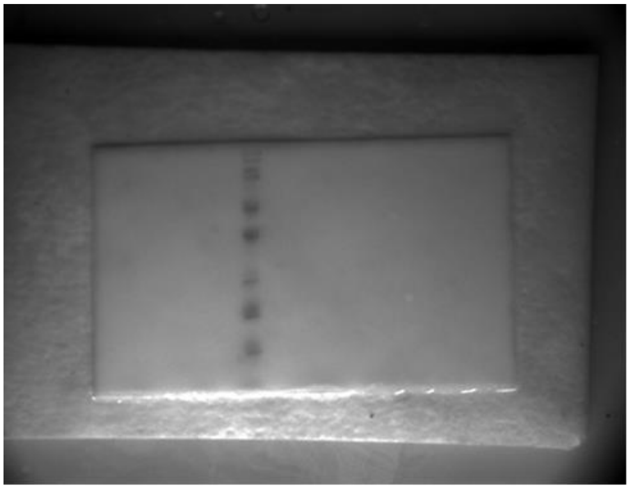

Membrane

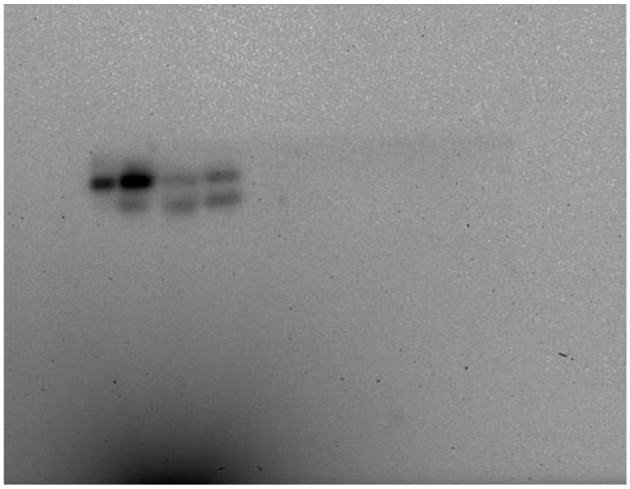

Anti-InlA mAb blot

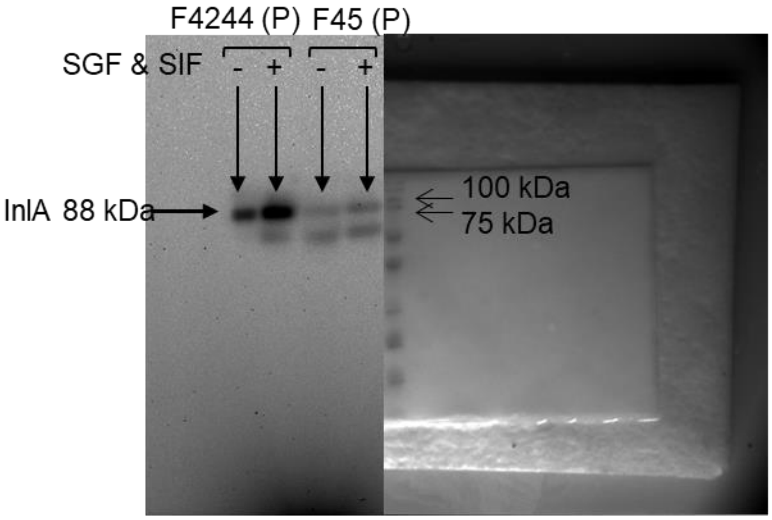

Merged

Figure 8e

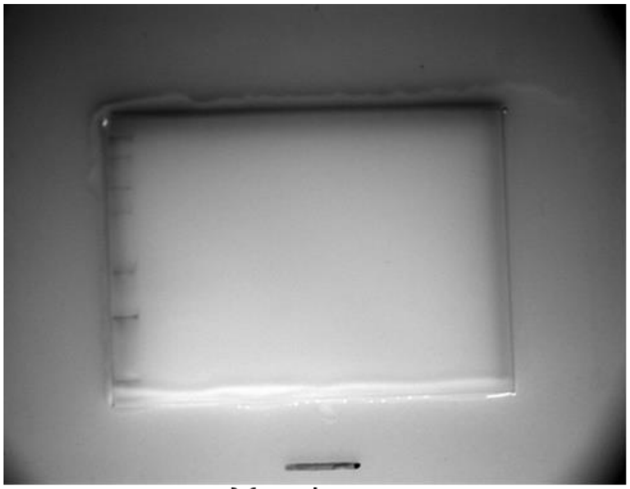

Membrane

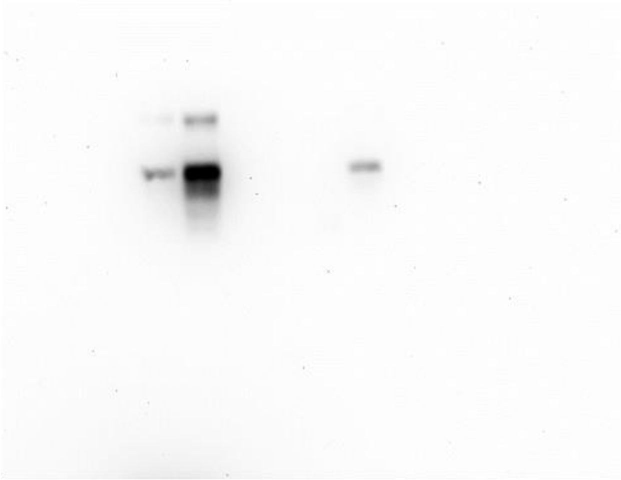

Anti-LAP mAb blot

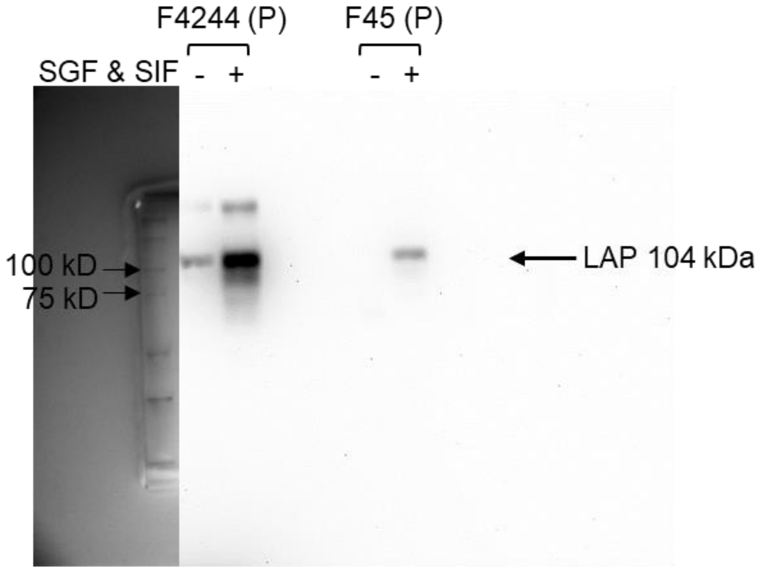

Merged

Figure 8e

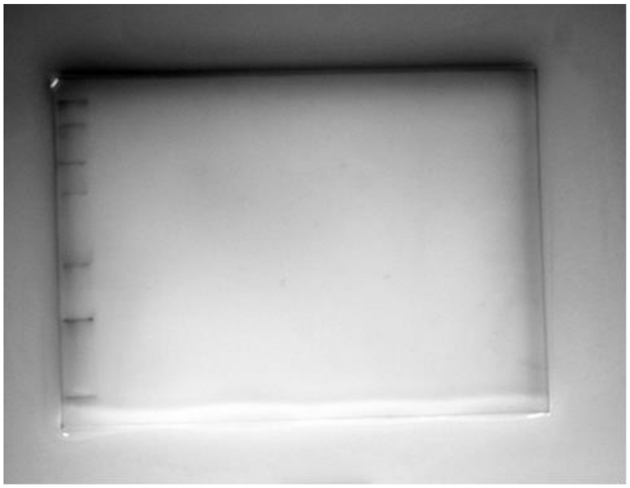

Membrane

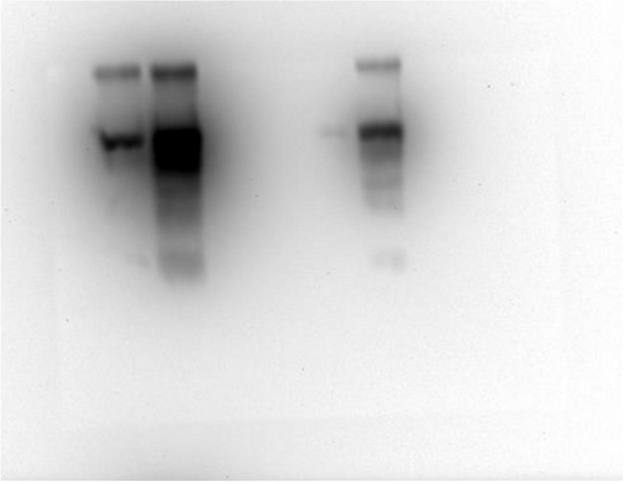

Anti-InlA mAb blot

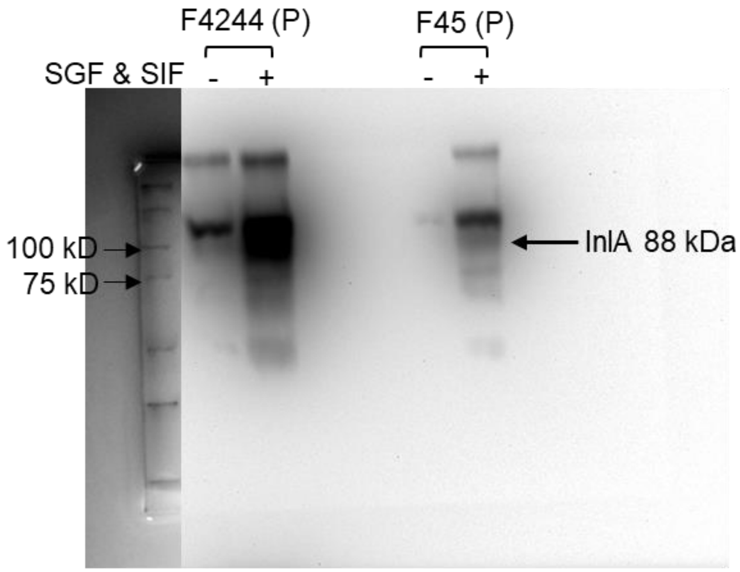

Merged

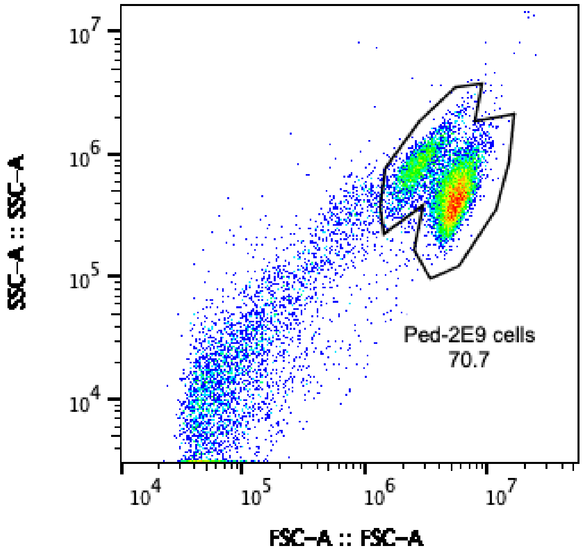

Plots exemplifying the gating strategy used for the flow cytometry analysis. Ped-2E9 cells were gated in FSC and SSC and analyzed for PE and 7-AAD.
