# Supplementary material for: Biofilm-isolated Listeria monocytogenes exhibits reduced systemic dissemination at the early (12–24 h) stage of infection in a mouse model
Source: NPJ Biofilms Microbiomes. 2021 Feb 8;7:18. doi: 10.1038/s41522-021-00189-5 (PMC7870835; doi:10.1038/s41522-021-00189-5)
Supplement: Supplementary file 2 — Reporting Summary [file 41522_2021_189_MOESM2_ESM.pdf]

nature research

Corresponding author(s): Professor Arun K. Bhunia

Last updated by author(s): 2020/11/13

Reporting Summary

Nature Research wishes to improve the reproducibility of the work that we publish. This form provides structure for consistency and transparency in reporting. For further information on Nature Research policies, see Authors & Referees and the Editorial Policy Checklist.

Statistics

For all statistical analyses, confirm that the following items are present in the figure legend, table legend, main text, or Methods section.

n/a	Confirmed	
☐	☒	The exact sample size (*n*) for each experimental group/condition, given as a discrete number and unit of measurement
☐	☒	A statement on whether measurements were taken from distinct samples or whether the same sample was measured repeatedly
☐	☒	The statistical test(s) used AND whether they are one- or two-sided<br>*Only common tests should be described solely by name; describe more complex techniques in the Methods section.*
☒	☐	A description of all covariates tested
☐	☒	A description of any assumptions or corrections, such as tests of normality and adjustment for multiple comparisons
☐	☒	A full description of the statistical parameters including central tendency (e.g. means) or other basic estimates (e.g. regression coefficient) AND variation (e.g. standard deviation) or associated estimates of uncertainty (e.g. confidence intervals)
☐	☒	For null hypothesis testing, the test statistic (e.g. *F*, *t*, *r*) with confidence intervals, effect sizes, degrees of freedom and *P* value noted<br>*Give P values as exact values whenever suitable.*
☒	☐	For Bayesian analysis, information on the choice of priors and Markov chain Monte Carlo settings
☒	☐	For hierarchical and complex designs, identification of the appropriate level for tests and full reporting of outcomes
☒	☐	Estimates of effect sizes (e.g. Cohen's *d*, Pearson's *r*), indicating how they were calculated

Our web collection on statistics for biologists contains articles on many of the points above.

Software and code

Policy information about availability of computer code

Data collection	Leica Application Suite version X software was used to collect fluorescence and brightfield images. BD Accuri C6 was used to collect flow cytometry data.
Data analysis	Microscoft Excel 2010/365, ImageJ 1.51t, Graphpad Prism 6.0, SnapGene, FlowJo, Bio Rad Quantity One, Leica Application Suite version X.

For manuscripts utilizing custom algorithms or software that are central to the research but not yet described in published literature, software must be made available to editors/reviewers. We strongly encourage code deposition in a community repository (e.g. GitHub). See the Nature Research guidelines for submitting code & software for further information.

Data

Policy information about availability of data

All manuscripts must include a data availability statement. This statement should provide the following information, where applicable:

- Accession codes, unique identifiers, or web links for publicly available datasets
- A list of figures that have associated raw data
- A description of any restrictions on data availability

All requests for raw and analyzed data and materials are promptly reviewed by to verify if the request is subject to any intellectual property or confidentiality obligations. Any data and materials that can be shared will be released via a Material Transfer Agreement.

Field-specific reporting

Please select the one below that is the best fit for your research. If you are not sure, read the appropriate sections before making your selection.

☒ Life sciences ☐ Behavioural & social sciences ☐ Ecological, evolutionary & environmental sciences

For a reference copy of the document with all sections, see nature.com/documents/nr-reporting-summary-flat.pdf

Life sciences study design

All studies must disclose on these points even when the disclosure is negative.

Sample size	Sample sizes were based on previous experience with these types of experiments. The analysis after each experiment verifies that these were sufficient
Data exclusions	No data were excluded
Replication	All data are representative of two or more independent experiments. Specifics are provided in each figure legend. All attempts at replication were successful.
Randomization	Randomization was used for mouse infections by using mice that were chosen randomly. For in vitro studies, randomization was not necessary, as the results were not qualitative.
Blinding	Investigators were blinded during quantitative measurements of imaging samples and histological analyses.

Reporting for specific materials, systems and methods

We require information from authors about some types of materials, experimental systems and methods used in many studies. Here, indicate whether each material, system or method listed is relevant to your study. If you are not sure if a list item applies to your research, read the appropriate section before selecting a response.

Materials & experimental systems

n/a	Involved in the study	
☐	☒	Antibodies
☐	☒	Eukaryotic cell lines
☒	☐	Palaeontology
☐	☒	Animals and other organisms
☒	☐	Human research participants
☒	☐	Clinical data

Methods

n/a	Involved in the study	
☒	☐	ChIP-seq
☐	☒	Flow cytometry
☒	☐	MRI-based neuroimaging

Antibodies

Antibodies used	All antibodies used have been listed with the catalog numbers in the supplementary table 4.<br>1. Mouse monoclonal anti-LAP Our Lab N/A<br>2. Mouse monoclonal anti-InlA antibody (mAb-2D12), our Lab, N/A<br>3. Rabbit polyclonal anti-LLO antibody Cat # ab200538; Abcam, Cambridge, UK
Validation	Mouse monoclonal anti-LAP, and anti-InlA antibodies have been validated in our lab by western blot against recombinant LAP, and InlA, respectively and listeria whole-cell lysates and showing that it only reacts with LAP and InlA, respectively (Drolia et al. Cell Host Microbe, 2018) (Burkholder & Bhunia. Infection and Immunity, 2010). Anti-LLO antibody was purchased from a vendor who have already validated its performance.

Eukaryotic cell lines

Policy information about cell lines

Cell line source(s)	The human colon carcinoma Caco-2 cell line (ATCC # HTB37) from 25-35 passages were used.<br>The human ileocecal HCT cell line was purchased from ATCC.
Authentication	The cell line was authenticated on the basis of morphology, basal barrier function, and loss of barrier function by cytokines.

Mycoplasma contamination	Cells were routinely tested for mycoplasma contamination and were negative throughout the course of these studies.
Commonly misidentified lines (See ICLAC register)	None Used

Animals and other organisms

Policy information about studies involving animals; ARRIVE guidelines recommended for reporting animal research

Laboratory animals	Black mice (C57BL/6: 8-10 weeks of age, male and female) from our breeding colony
Wild animals	None Used
Field-collected samples	None Used
Ethics oversight	The animal procedure was approved by the Purdue University Animal Care and Use Committee (PACAUC approval No.1201000595) who adheres to the recommendations of the Guide for the Care and Use of Laboratory Animals published by the National Institute of Health.

Note that full information on the approval of the study protocol must also be provided in the manuscript.

Flow Cytometry

Plots

Confirm that:

☒ The axis labels state the marker and fluorochrome used (e.g. CD4-FITC).

☒ The axis scales are clearly visible. Include numbers along axes only for bottom left plot of group (a 'group' is an analysis of identical markers).

☒ All plots are contour plots with outliers or pseudocolor plots.

☒ A numerical value for number of cells or percentage (with statistics) is provided.

Methodology

Sample preparation	Freshly grown Ped-2E9 cells were counted by Trypan blue staining and resuspended in 500 µl D10F with the final cell concentration of approx. After Ped-2E9 cells were treated with bacteria, Ped-2E9 cells were stained with Annexin V-PE and 7-AAD (BD, Franklin Lakes, NJ) following the vendor's protocol. Specifically, cells were centrifuged down at 57 RCF for 3 min (Hettich), rinsed twice with cold PBS, and resuspended in 1X binding buffer at concentration of approx. 1 million cell/ml. One hundred microliter of the cell suspension was added with 5 µl FITC Annexin V and 5 µl PI. The suspension was gently mixed and incubated in ambient temperature for 15 min in the dark. After staining, 400 µl 1X binding buffer was added into the cell ßsuspension before analysis by flow cytometry.
Instrument	BD Accuri™ C6 (BD, Franklin Lakes, NJ)
Software	BD Accuri™ C6 software, FlowJo
Cell population abundance	At least 10,000 events were collected from each sample
Gating strategy	Ped-2E9 cells were gated in FSC and SSC and analyzed for PE and 7-AAD staining.

☐ Tick this box to confirm that a figure exemplifying the gating strategy is provided in the Supplementary Information.
